# Defective quality control autophagy in Hyperhomocysteinemia promotes ER stress and consequent neuronal apoptosis through proteotoxicity

**DOI:** 10.1186/s12964-023-01288-w

**Published:** 2023-09-25

**Authors:** Bhavneet Kaur, Pradeep Kumar Sharma, Barun Chatterjee, Bhawana Bissa, Vasugi Nattarayan, Soundhar Ramasamy, Ajay Bhat, Megha Lal, Sarbani Samaddar, Sourav Banerjee, Soumya Sinha Roy

**Affiliations:** 1 CSIR-Institute of Genomics & Integrative Biology, Mathura Road, Sukhdev Vihar, New Delhi, 110020 India; 2 Academy of Scientific & Innovative Research, Ghaziabad, 201002 India; 3 Present Address: CSIR-Indian Institute of Toxicology Research, Lucknow, India; 4 Present address: Department of Biochemistry, School of Life Sciences, Central University of Rajasthan, Ajmer, India; 5 National Brain Research Centre, Manesar, India

**Keywords:** Homocysteine, Hyperhomocysteinemia, CBS, Neuron, Autophagy, Endoplasmic reticulum, Mitochondria, Apoptosis

## Abstract

Homocysteine (Hcy), produced physiologically in all cells, is an intermediate metabolite of methionine and cysteine metabolism. Hyperhomocysteinemia (HHcy) resulting from an in-born error of metabolism that leads to accumulation of high levels of Hcy, is associated with vascular damage, neurodegeneration and cognitive decline. Using a HHcy model in neuronal cells, primary cortical neurons and transgenic zebrafish, we demonstrate diminished autophagy and Hcy-induced neurotoxicity associated with mitochondrial dysfunction, fragmentation and apoptosis. We find this mitochondrial dysfunction is due to Hcy-induced proteotoxicity leading to ER stress. We show this sustained proteotoxicity originates from the perturbation of upstream autophagic pathways through an aberrant activation of mTOR and that protetoxic stress act as a feedforward cues to aggravate a sustained ER stress that culminate to mitochondrial apoptosis in HHcy model systems. Using chemical chaperones to mitigate sustained ER stress, Hcy-induced proteotoxicity and consequent neurotoxicity were rescued. We also rescue neuronal lethality by activation of autophagy and thereby reducing proteotoxicity and ER stress. Our findings pave the way to devise new strategies for the treatment of neural and cognitive pathologies reported in HHcy, by either activation of upstream autophagy or by suppression of downstream ER stress.

Video Abstract

**Supplementary Information:**

The online version contains supplementary material available at 10.1186/s12964-023-01288-w.

## Introduction

**Fig. 1:**
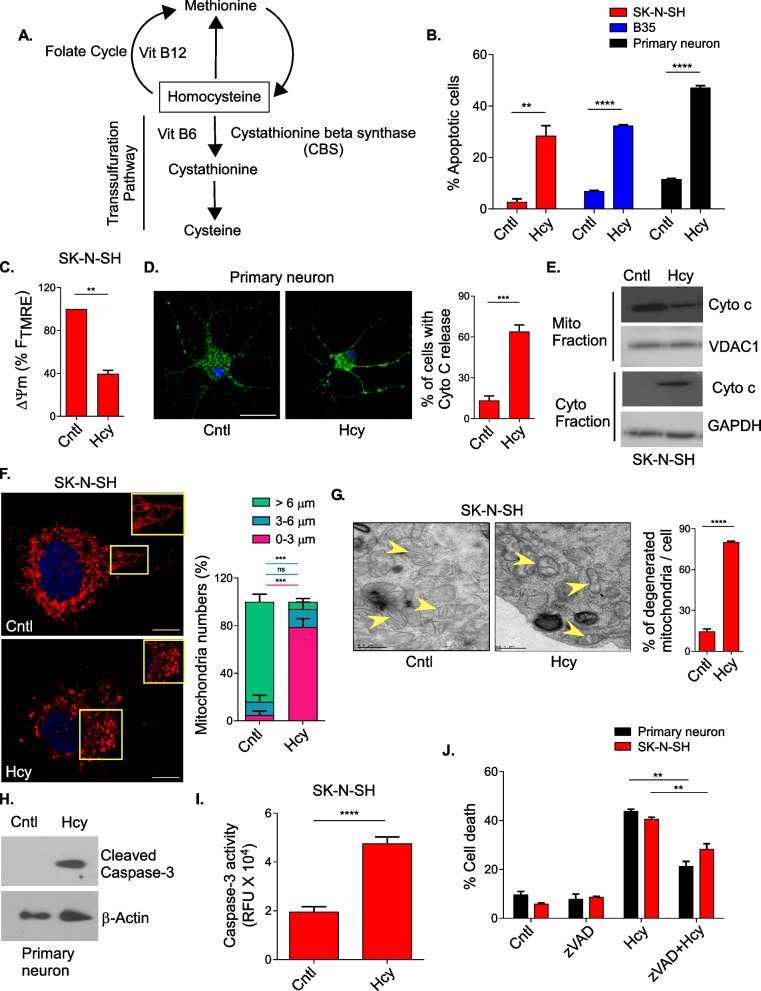
Hcy-induced neuronal toxicity is associated with mitochondrial dysfunction and apoptosis. **A** Simplified diagram showing Hcy as an intermediate of Met and Cys metabolism. **B** Bar plot showing percentage of apoptotic cell death in SK-N-SH (human neuroblastoma), B35 (rat neuroblastoma) and rat primary cortical neurons upon Hcy (5 mM) treatment for 24 h. **C** Loss of mitochondrial membrane potential (ΔΨm) in Hcy treated SK-N-SH cells as measured by potentiometric TMRE probe (500 nM). **D** Representative confocal images showing the release of Cytochrome c (Cyto c) from mitochondria in cyto c-GFP expressing primary cortical neurons treated with Hcy. Scale bar 20 µm. Bar graph represents the percentage of neurons showing Cyto c release upon Hcy treatment. **E** Representative immunoblot showing Cyto c level in isolated mitochondrial and cytosolic fraction of SK-N-SH cells post Hcy treatment. **F** Confocal images showing high level of mitochondrial fragmentation in mito-DsRed expressing SK-N-SH cells treated with Hcy. Insets showing zoomed portions of the images and bar graph showing the average length distribution of different mitochondrial populations. Scale bar 10 µm. **G** Representative transmission electron micrograph showing distorted mitochondrial structures with disrupted cristae in SK-N-SH cells treated with Hcy. Arrowheads represent mitochondria. Scale bar 0.5 µm. Bar graph showing the quantitation of the TEM images representing the percentage of damaged mitochondrial population per cell. **H** Representative immunoblot showing activated caspase-3 in primary neurons upon Hcy treatment. β-actin was used as a loading control. **I** Bars showing caspase-3 activation by measuring its enzymatic activity using AcDEVD substrate in Hcy treated SK-N-SH cells. **J** Pan caspase inhibitor zVAD-FMK (5 µM) rescued Hcy-induced toxicity in both primary cortical neurons and SK-N-SH cells. Concentration of Hcy was 5 mM for cell lines and 0.75 mM for primary neurons. Data are shown as Mean ± SEM with *n* ≥ 3. **P < 0.01. ****P < 0.0001

Homocysteine (Hcy) is a non-proteinogenic, thiol-containing alpha amino acid and is an important intermediate of methionine (Met) and cysteine (Cys) metabolism. It is synthesized from Met by S-adenosylmethionine mediated trans-methylation reactions and gets converted back to Met through re-methylation and to Cys through the transsulfuration pathway using B-vitamins as cofactors (Fig. 1A). Genetic defects in these pathways or nutritional deficiencies of folate and B-vitamins lead to elevation of cellular and plasma Hcy which is considered toxic to the cells and this abnormal increase of Hcy are conditions commonly known as Hyperhomocysteinemia (HHcy) [1]. There is strong evidence from clinical and laboratory studies that HHcy is an independent risk factor for several pathologies like cardiovascular diseases, endothelial dysfunction and chronic kidney failures [2]. Several animal model studies from the last two decades and growing epidemiological evidence of association of HHcy with age-related cognitive impairment, neurodegenerative and cerebrovascular diseases have increased the interest in the role of Hcy in neurological and neuropsychiatric disorders [3]. It is believed that excess intracellular generation of Hcy may cause central nervous system dysfunction through its neurotoxic properties. Supporting this claim, HHcy has been reported to increase the risk of Alzheimer’s disease (AD) [4, 5], Parkinson’s disease [5, 6], Multiple Sclerosis [7], Neural tube defect [8], Bipolar disorder [9], stroke [10] etc. as well as cochlear [11] and retinal dysfunction [12]. In addition, maternal HHcy has been rendered as one of the major complications in pregnancy to cause different functional impairments of the offspring brain leading to the decrease in memory and locomotors activity [13, 14]. Despite the well-established role of HHcy in an array of neurological disorders, the molecular consequences of high cellular levels of Hcy in neurotoxicity are largely unknown and often conflicting.

One of the causes of Hcy toxicity in different types of cell including neurons involves endoplasmic reticulum (ER) stress [15–17]. Disturbance of protein folding in pathological conditions causes increased load of misfolded and aggregated proteins, altered cellular redox or aberrant Ca^2+^ signaling thus creating ER stress and activating unfolded protein response (UPR) [18]. UPR activation is an adaptive mechanism for maintaining cellular homeostasis through induction of chaperone gene expression, suppression of global protein synthesis and activation of the proteasome and autophagy [19]. Failure in restoration of ER homeostasis leads to induction of C/EBP homologous protein (CHOP), a downstream pro-apoptotic transcription factor that triggers intrinsic apoptosis [20]. As ER stress is a hallmark of several neurodegenerative disorders [21], its role in HHcy-induced neurotoxicity could well be a speculation. However, how excess Hcy evokes ER stress in neurons is not yet clearly defined.

Hcy toxicity in neuronal and other cells has frequently been linked with mitochondrial dysfunction [22] but whether it is a direct consequence of upstream ER stress is not clear. Most of the existing literature suggest the role of excess ROS or Ca^2+^ overload in mitochondrial dysfunction and apoptosis during HHcy, although several studies antagonize these claims. Many reports indicate an increased ROS generation and oxidative damage [23–25] whereas several other studies show a decrease in both ROS level and oxidative damage [26, 27] upon Hcy treatment. Several reports also indicate that Hcy-mediated neuronal apoptosis is by excitotoxicity through N-methyl-D-aspartate (NMDA) receptor activation that culminates in Ca^2+^ overload dependent mitochondrial permeabilization [27, 28]. However, recent studies confirmed that Hcy-induced neurotoxicity is different from excitotoxic cell death and it is less dependent on calcium imbalance [29, 30]. These opposing reports suggest that alternative mechanisms of Hcy neurotoxicity need to be considered.

Apart from apoptosis, the other cell death process that influences neurons and eventually causes neurodegeneration is autophagy [31, 32]. Autophagy is a major cellular degradation pathway that eliminates damaged cellular components like proteins, lipids and other organelles thus maintains cellular homeostasis [33, 34]. It is well documented that autophagy plays an important role in neurodegeneration and it is activated during ER stress to maintain protein homeostasis and neuronal survival [35, 36]. However, autophagy can also cause mitochondrial dysfunction and apoptosis through Beclin1 and other pro-apoptotic proteins [37, 38]. Though HHcy has been shown to induce ER stress and apoptosis, its effect on autophagy is not well documented. Couple of reports describe a decrease [39, 40] whereas several studies show an increase [41, 42] in autophagy during HHcy. Thus the role of autophagy in Hcy-induced toxicity is an important area that requires further elucidation.

As ER stress-induced UPR-activation also maintains cellular homeostasis through crosstalk with autophagy and mitochondrial functionality to determine cell fate, studies on the functional interrelation of these three processes in different pathologies is attracting immense importance [43]. Because the mechanisms of neurotoxicity during HHcy is unclear, and as alteration of all these three cellular processes are reported in Hcy-induced toxicity, we examined whether the neuronal lethality resulting from HHcy is mediated by perturbation of fine-tuned crosstalk among those pathways. In three model systems – neuronal cell lines, cultured primary cortical neurons and transgenic *zebrafish* – we find that HHcy causes neuronal apoptosis by sequential perturbation of autophagy, ER stress and mitochondrial dysfunction. We also demonstrate that mitochondrial dysfunction is not due to oxidative stress or mitochondrial Ca^2+^ overload, rather due to proteotoxicity. Importantly, we rescue this neuronal lethality independently by both the upstream activation of autophagy and downstream blocking of ER stress using chemical chaperones. Reanalyzing the transcriptomic data of a transgenic viable hyperhomocysteinemic *Drosophila* model, we also show that HHcy-induced systemic autophagic perturbation is counterbalanced by intrinsic activation of the stress response pathway for its survival and that further strengthen our experimental results.

## Materials and methods

### Cell culture, transfection and stable cell line generation

Cell culture media and reagents were purchased from Thermo Fisher unless otherwise mentioned. Neuroblastoma cells SK-N-SH (Human), Neuro-2a (mouse) and B35 (rat) were purchased from ATCC. All three neuroblastoma cells were maintained in DMEM, supplemented with 10% FBS and 100 units of Antibiotic–Antimycotic solution. Primary dissociated cultures of cortical neurons were prepared following the published protocol [44] with slight modifications. The cortices of E18 embryos harvested from pregnant Sprague Dawley rats and the dissociated cortical neurons were seeded on poly-L-lysine (1 mg/ml) coated 18 mm coverslips at 25,000 cells/sq.cm. and on pre-coated 60 mm dishes (BD Biosciences) at 13000–15000 cells/sq.cm. These cultures were maintained in Neurobasal medium with B-27 supplement till DIV 7–9. To prevent the growth of glial cells,  cytosine arabinoside (Ara-C) was added at a concentration of 1.5 μM on DIV-3. Ara-C was replenished every seven days in the culture media, till the time of harvest. For stable cell generation in neuroblastoma lines, cells were transfected with GFP-LC3 and mitoDsRed plasmids using lipofectamine 2000 and Opti MEM medium as per manufacturer’s instructions. Plasmids (500 ng) were mixed with lipofectamine 2000 (1:2 ratio) and left to stand for 20 min. After 4–6 h post transfection, culture media was replaced with fresh complete growth media. 48 h post-transfection, cells were treated with G418 (Sigma) initially at a concentration of 100 μg/ml. After two days the culture media was changed with a growth medium without G418 to allow them to grow further. The exposure of G418 was repeated to a final concentration of 1 mg/ml till 80–90% cells were positive for the expression of the plasmid. For the selection of stable clones, a single cell suspension of 100 cells/60 mm dish were seeded and allowed them to form colonies. Every colony was screened for GFP or DsRed signal under fluorescence microscope and the single positive colony with highest signal intensity was selected and cultured routinely in a growth medium containing 50 μg/ml of G418. These cells were used for further experiments. Primary cortical neurons were transiently transfected with GFP-LC3 and mitoDsRed plasmids using lipofectamine 2000 and Opti-MEM medium as per manufacturer’s instruction. Cells were cultured in a humidified atmosphere under standard growth condition of 37 °C with 5% CO_2_. Identity authentication of SK-N-SH cells were carried out by STR profiling (Lifecode Technologies) and mycoplasma testing was carried out regularly using a mycoplasma detection kit (Lonza).

### Zebrafish maintenance and morpholino microinjection

Wild type zebrafish and stable transgenic line Tg(HuC:Kaede), which was obtained from RIKEN [45], was bred, raised and maintained at 28.5 °C on a 14 h light/10 h dark cycle and were housed at the CSIR – Institute of Genomics and Integrative Biology (IGIB), Mathura Road, New Delhi, India. All experiments were approved by the Institutional Animal Ethics Committee (IAEC). Morpholinos were synthesized by Gene Tools (Philomath, OR, USA). For knockdown of Cystathionine-β-synthase (CBS), a cocktail of translation blocking morpholinos against CBSa and CBSb genes were used along with a 5-base mismatch control morpholino. 8 ng of each of the morpholinos was injected into 1-cell stage embryos at a constant volume of 3 nl. Microinjection was done in a PLI-90 Pico-Injector (Harvard Apparatus) equipped with a Narishige micromanipulator. Glass capillary needles (World Precision Instruments) were pulled using Sutter Instrument (USA) and for every microinjection, needles were calibrated using Drummond Microcaps (Sigma, Cat No. P1424). Following morpholino injections, embryos were placed in E3 medium in 90 mm Petri dishes at a density of 80 embryos per dish. Embryos were raised at 28 °C till 24 hpf. The sequences of the morpholinos were as follows:

- CBSa MO: 5’-GGGACTGAAGGCATTATTCCTCAAT-3’:
- CBSb MO: 5’-CTGGCATGGTTTACCCTGACTATCA-3’:
- Cntl MO: 5’-GGCACTCAAGGCAATATACGTCAAT-3’.

### Lentiviral transduction

Lentiviral particles were generated using Lenti-X TM 293 T cell line (Clontech). 3rd generation lentiviral packaging plasmids, pRSV-Rev (addgene ID 12253), pMDLg/pRRE (addgene ID 12253) and pMD2.G (addgene ID 12259), were used. Lenti HEK 293 cells were plated 24 h before transfection and transfected with the plasmid cocktail (packaging and transfer plasmids) using Lipofectamine 2000. Briefly plasmid cocktails were transfected using lipofectamine in 1:2 ratio in Opti-MEM media; this medium was then replaced by normal growth medium 4 h post transfection. Media containing viral particles were harvested twice, once 48 h post transfection and the second time, 72 h post transfection. This media was first cleared by low speed centrifugation and then filtered through a 0.45 μm pore size polyethersulfone membrane. To calculate multiplicity of infection, firstly, the lentiviruses were titrated by qPCR based lentiviral titration kit (LV900, ABM) followed by counting positive colonies for respective titration volumes. Monolayer cultures of SK-N-SH and B35 cells (5 × 10^5^ cells/ml) grown in a 6-well plate were infected with lentiviral vectors at a multiplicity of infection of 10 for 24 h followed by a change of media. The cells were incubated for another 2 days and then analyzed for ATG5 and ATG7 overexpression.

### Antibodies and reagents

The following antibodies were used for performing western blotting: anti-Beclin1 (CST 3495, 1:1000), anti-ATG5 (CST 2630, 1:1000), anti-ATG7 (CST 8558, 1:1000), anti-LC3B (CST 12741, 1:1000), anti-p62 (BD 610833, 1:1000), anti-BiP (BD 610979, 1:2000), anti-CHOP (CST 2895, 1:1000), anti-IRE1p (Enzo ADI-905–727-100, 1;1000), anti-mono and polyubiquitinylated conjugates (Enzo BMLPW8810, 1:1000), anti-GAPDH (CST 5174, 1:2000), anti-Actin (BD 612657, 1:2000), anti-⍺-Tubulin (CST 2125, 1:2000), anti-Phospho-mTOR (CST 2974, 1:1000), anti-mTOR (CST 2983, 1:1000), anti-Phospho-p70 S6K (CST 9205, 1:1000), anti-p70 S6K (CST 9202, 1:1000), anti-phospho-4EBP1 (CST 2855, 1:1000), anti-4EBP1 (CST 9644, 1:1000) and for zebrafish, anti-Beclin1 (Abcam ab207612, 1:1000) and anti-p62 (Abcam ab56416, 1:1000). Horseradish peroxidase (HRP) conjugated anti-rabbit (GE Healthcare, NA934) and anti-mouse (GE Healthcare, NA931) secondary antibodies were used. RIPA lysis buffer (Pierce, 89,901) and for zebrafish NP-40 lysis buffer (Thermo Fisher, FNN0021) with protease inhibitor cocktail (Roche, 04693159001) and phosphatase inhibitor cocktail (Roche, 4,906,845,001) for lysate preparation. Homocysteine (H4628), Trypan blue (T8154), CSA (30,024), RuRed (R2751), 4-PBA (P21005), DiOC6 (318,426), NAC (A7250), FCCP (C2920) and TUDCA (T0266) were from Sigma. Rhod 2AM (R1244), TMRE (T669), MitoSOX Red (M36008), DCFDA (C6827), Propidium iodide (P3566), Annexin V-Alexa fluor 488 (A13201), annexin binding buffer (V13246) were from Invitrogen and Wortmannin (BML-ST415-0005), Z-VAD-FMK (ALX-260–020-M005), Rapamycin (BML-A275-0025), Bafilomycin A1 (ALX-380–030) were from Enzo.

### Lysate preparation

Equal number of cells were incubated in RIPA buffer, supplemented with protease inhibitor cocktail and phosphatase inhibitor cocktail for 30 min at 4 °C and then centrifuged at 10,000 rpm for 20 min at 4 °C. Same protocol was followed for zebrafish embryos (with equal number of embryos) except that embryos were homogenized in the NP-40 lysis buffer for about 10–20 s at 800 rpm before incubation for 30 min. After incubation, it was centrifuged at 10,000 rpm for 20 min at 4 °C. After centrifugation, the supernatant was used as lysate.

### Western blotting

Lysates were boiled in 5X Laemmli Buffer for 10 min before loading. An equal concentration of protein (30 μg) was resolved by 8–12% SDS-PAGE and then transferred onto 0.2 μm PVDF membrane. The membranes were blocked with 5% BSA for 2 h at room temperature followed by an overnight incubation in respected primary antibodies at 4 °C. Membranes were washed thrice (for 15 min each time) with TBS containing 0.1% tween and then incubated with HRP-conjugated secondary antibody (GE, 1:10,000 dilution) at room temperature for 2 h. Proteins were detected using the Immobilon chemiluminescent substrate for HRP (Millipore) and images were analyzed by chemiluminescent image analyzer (G:Box). Quantitation was performed by ImageJ software.

### Homocysteine measurement

Intracellular Hcy concentration was detected using HPLC. Neuroblastoma cells were harvested by trypsinization and were washed with 1X PBS. Cells were lysed in RIPA buffer and 100 ul of the cell lysate was treated with 35 μl of 1.43 M sodium borohydride in 0.1 M NaOH (to reduce disulphide bonds). Then 10 μl of amyl alcohol was added to avoid foaming, followed by addition of 35 μl of 1 M HCL. To this mixture, 50 μl of 7 mM bromobiamine in 5 mM sodium EDTA (pH 7.0) was added (to conjugate the reduced thiols with flurophore) and the solution was incubated at 42 °C for around 15 min followed by incubation at RT for another 30–45 min. 50 μl of 1.5 M perchloric acid was used to precipitate intracellular proteins followed by centrifugation at 12,000 rpm for 10 min; the supernatant was neutralised with 6 μl of 2 M Tris base. From this, 4 μl of derivatized sample was injected into a 100 × 4.6 mm, 1.8 micron Eclipse plus C18 column using Agilent-1260 Ultra-high performance liquid chromatography (UPLC). Column was equilibrated with 90% buffer A (Composed of 5% methanol and 0.86% acetic acid in water) and 10% Buffer B (100% methanol). Thiols were eluted from the column with linear gradient of both buffers (from 90% buffer A, 10% buffer B to 0% buffer A and 100% buffer B) in 7 min. Flow rate was maintained at 0.7 ml/min at RT. Standard curve was generated with known concentrations of Hcy. Results were quantified by taking the area for the Hcy-bimane peak and calculating its concentration using a regression equation derived from a standard curve. For zebrafish samples, 24 hpf embryos were lysed in NP-40 buffer and 100 ul of the lysate was used for subsequent processing.

### Imaging analysis

For confocal imaging, SK-N-SH and B35 cells stably expressing GFP-LC3 and DsRed were grown on coverslips and next day pretreated with various inhibitors and inducers of autophagy and ER stress (as indicated in results section). Following treatment with Hcy, cells were washed with cold PBS (1X) and fixed with 4% paraformaldehyde for 10–20 min at room temperature. Coverslips were then mounted onto glass slides using DAPI containing mounting solution (Invitrogen) and sealed. These fixed cells were imaged at 63X using oil immersion objective of a laser scanning confocal microscope (LSM510 META, Ziess) equipped with a camera. Suitable excitation and emission band pass filters for GFP-LC3 and DsRed were used to design light path and images were captured. For the quantification of GFP-LC3 puncta at single cell level, cells were counted and cells with more than 10 puncta were considered positive. At least 50 cells were counted for three different experiments. Mitochondrial length analysis were done using Fiji package in ImageJ2.

For imaging, zebrafish embryos at 24 hpf were embedded in 2.5% methylcellulose and imaged using a Nikon SMZ800N (ED Plan 0.75X objective) stereo-microscope at 5X. Fluorescence images were taken using ZEISS Axioskop 40 microscope at 5X. Confocal microscopy was performed with Nikon AR1 confocal microscopy with plan apo 10X objective.

### Immunofluorescence analysis

Cells grown on coverslips were fixed using fixing solution (37% formaldehyde and 0.2% Glutaraldehyde in TBS) for 20 min at 4 °C. Cells were then permeabilized with 0.1% Triton X-100 for 10 min at 4 °C. After that, blocking was done with 2% BSA for 2 h at room temperature. This was followed by overnight primary antibody (1:250 in 1% BSA) incubation at 4 °C. After that, cells were washed thrice with Tris-buffered saline (TBS) for 15 min each. Thereafter, alexa fluor 647 conjugated secondary antibody (1:500 in 1% BSA) (Thermo A21244) was added to the cells for 2 h at room temperature. Finally, three washes of 15 min each were given to the cells followed by mounting with Prolong Gold mounting solution with DAPI (Thermo P36935). Confocal images were taken at 63X objective.

### Transmission electron microscopy

For electron microscopy, SK-N-SH cells were fixed with 2.5% glutaraldehyde and 4% paraformaldehyde in 0.1 M phosphate buffer (pH 7.4) overnight. The samples were osmicated with 1% osmium tetroxide post fixation and then dehydrated in graded series of alcohol and infiltrated with Epon 812 resin. Ultrathin sections were cut on RMC ultramicrotome, collected on copper grids and stained with uranyl acetate and lead citrate. Samples were visualized on Tecnai G2 20 twin (FEI) transmission electron microscope. Morphologic analyses of the TEM images were performed manually on a sample of at least 25 randomly selected images in each of the control and the Hcy-treated groups. Healthy mitochondrial population was selected by their dense matrix with tightly packed granules and well-defined cristae structures. Whereas, damaged mitochondria were selected by their swelled and fragmented structures with less dense matrix and disrupted cristae structures. Autophagosomes were selected by their double-membraned vesicular structures, a well-defined and characteristic feature of autophagic vesicles.

### Cell viability assays

For trypan blue exclusion assay, cells were seeded at a density of 5 × 10^4^ per well in 24-well flat bottom culture plates and treated next day with the indicated concentration of Hcy for 24 h. Following treatment, cells were collected (including floaters, which are dead cells) and stained with trypan blue (0.4% solution) for 2 min. Live cells (trypan negative) and dead cells (trypan positive) were counted with the help of hemocytometer and cell death were represented as percentage of dead cells. For primary cortical neurons, 4 X 10^4^ cells were seeded onto coverslips for seven days. Then cells were treated with the indicated concentration of Hcy for 24 h. Following treatment, cells were stained with Propidium iodide (PI) for 10 min. Stained cells then fixed with 4% PFA (paraformaldehyde) for 15–20 min and mounted onto glass slides using DAPI containing mounting solution. Slides were imaged and representative images were captured. PI positive cells were counted in at least 10 different fields per experiment (*n* = 3).

### Apoptotic cell detection

Apoptosis detection was done using Alexa fluor-488 and PI dual staining method. Both the neuroblastoma cells from human and rat origin were seeded onto 6-well plates at a density of 1 × 10^5^ cells per well. Next day cells were treated with 5 mM concentration of Hcy for 24 h. Following treatment, cells were harvested by trypsinization and washed twice with PBS. Then the harvested cells were suspended in Annexin V binding assay buffer and labeled with Alexa fluor-488 and PI for 15 min as per manufacturer’s protocol. Neuroblastoma cells were then analyzed by flow cytometry (BD Bioscience) and a minimum of 10,000 cells were collected per sample. Early and late apoptosis was determined by using bi-variant plot analysis and data was represented as percentage of apoptotic (early + late) cell death. In the case of primary neurons, 40,000 cells grown on cover glass were treated with Hcy as indicated above. Post treatment, cells were labeled with Annexin V-Alexa fluor 488 antibody and PI. Images were captured at 40X objective under epifluorescence microscope using suitable emission filters. To quantify apoptotic cell death in primary neurons, at least 10 different fields were counted per group per experiment (*n* = 3).

For zebrafish embryos, apoptotic cells were detected by TUNEL assay using In Situ Cell Death Detection Kit (Fluorescein, Roche) according to manufacturer’s protocol. Briefly, Embryos were fixed with 4% paraformaldehyde, overnight at 4 °C, followed by gradual dehydration (30%, 50%, 70%, 100%) with methanol and kept at -20 °C overnight. Next day, embryos were gradually rehydrated and then permeabilized by treating them with 25 μg/ml proteinase K for 10 min at 30 °C and incubated with the TUNEL reaction mixture (labeling solution plus enzyme solution) at 37 °C for 2 h in a humidified atmosphere. This was followed by two washing with PBST, before imaging. Each sample contained approximately thirty embryos which were analyzed by Nikon Ti2-E confocal microscopy. For rescue experiments, in vitro translation (IVT) and microinjection of Beclin1 mRNA was done as per previously published protocol [46]. 250 pg of Beclin1 mRNA was co-injected with the CBS morpholino at 1-cell stage.

### Cytochrome c (Cyto c) release assay

Cyto c release from mitochondria was measured as previously described [47]. For imaging analysis, cells were transfected with Cyto c-GFP plasmid and later treated with Hcy for 24 h. Then coverslips were washed twice with PBS and transferred to the microscope stage for imaging the Cyto c-GFP distribution. For immunodetection, Hcy treated cells were harvested and washed with PBS. Equal number of cells were then re-suspended in 1 ml PBS containing 100 mM KCl and 50 µg/ml digitonin for permeabilization and it was done at 37 °C for 10 min with continuous stirring. Then the membrane and supernatant fractions were isolated rapidly by centrifugation at 10,000 g for 5 min, followed by immunoblotting.

### Mitochondrial calcium measurement

For detection of mitochondrial calcium level, Rhod-2AM staining was performed. Samples were subjected to Hcy treatment for 24 h following which the total fluorescence intensities of Rhod- 2AM was measured either by using a micro-plate reader (Tecan) for neuroblastoma cells or by a fluorescence microscope (Zeiss) for primary neurons.

### Mitochondrial membrane potential measurement

Mitochondrial membrane potential (ΔΨm) was examined using two potentiometric dyes—TMRE and DiOC6. The mean fluorescence intensities were measured by fluorescence plate reader and by microscope. Hcy treated neuroblastoma cells were harvested and washed with PBS and then incubated with TMRE (500 nM) for 15 min in the dark at 37 °C. For primary cortical neurons, 40,000 cells were grown in a 12-well culture plate as mentioned above. After that cells were treated with Hcy for 24 h and then labeled with DiOC6 (200 nM) for 30 min in the dark. After staining cells were washed with 1X PBS and fixed with 4% paraformaldehyde for 10–20 min. Coverslips were mounted onto glass slides and observed under the 40X objective of a fluorescence microscope (Nikon Eclipse) using suitable emission filters for the dyes. Images were captured using Nlite-Software. Quantitation of fluorescence intensity was performed using volocity software (Perkin Elmer) and at least 10 different images were quantified per group per experiment (*n* = 3).

### Measurement of reactive oxygen species (ROS)

Total and mitochondrial ROS levels were detected using the fluorescent dyes 2′,7′-dichlorodihydrofluorescein diacetate (DCFH-DA) and MitoSOX respectively. Cells were incubated with DCFH-DA (10 μM) and MitoSOX Red (5 μm) for 15 min in the dark, and the mean intensity of fluorescence was measured either by flow cytometry or by fluorescence microscopy (using 20X objective). Percentage of mean fluorescence intensities were calculated for representation.

### Caspase activity assay

Following treatment, cells were trypsinized and harvested and washed with 1X PBS. Cells were resuspended in cell lysis RIPA buffer and homogenized by intermittent vortexing and incubating on ice for 30 min. Cell lysates were centrifuged at 15,000 × g for 20 min at 4 °C and the supernatant was collected for total protein estimation using BCA method. A total of 100 μg protein from each sample was used for caspase-3 activity in the buffer containing Ac-DEVD-AMC substrate according to manufacturer’s protocol (Enzo life sciences).

### Source and preprocessing of fly expression data

The GSE148109 expression profiling dataset was used to determine the effect of deletion of cystathionine B-synthase (CBS) on autophagy and ER stress. The raw RNA-seq read counts of CBS8 deletion and control group were obtained for three biological replicates of whole female fly, *Drosophila melanogaster*. The data were normalized for sequencing depth and library composition using variance stabilizing transformation implemented in R package, DeSEQ2 [48]. The gene identifiers were matched with gene symbol using R package, org.Dm.eg.db (Marc Carlson (2020). org.Dm.eg.db: Genome wide annotation for Fly. R package version 3.12.0.). Genes related to autophagy pathway and heat-shock proteins were filtered for further analysis. Thereafter, the hierarchical cluster analysis based on Euclidean distance was performed using R package, pheatmap (Raivo Kolde (2019). pheatmap: Pretty Heatmaps. R package version1.0.12.).

### Statistical analysis

Until otherwise mentioned all the data are from 3 independent experiments. Results were expressed as mean ± SEM. Statistical significance between control and treated groups were tested by unpaired student’s t test. For more than two samples, one-way Anova was used. *P*-value < 0.05 was considered as significant.

## Results

### Hcy-induced neuronal toxicity is associated with mitochondrial dysfunction and apoptosis

We first assessed the effect of Hcy on survival of neuronal cells and for this purpose we treated a human neuroblastoma cell SK-N-SH with different doses of Hcy. Cell death assay by Trypan blue counting method showed a modest amount (almost 40%) of death upon treatment with 5 mM Hcy for 24 h (Figure S1A). To further check how much of 5 mM Hcy was entered inside the cell, we measured the intracellular level of Hcy in treated and untreated cells by HPLC. It showed that the intracellular concentration of Hcy was in micromolar range and there was a eightfold increase in Hcy level in the cells treated with 5 mM Hcy (Figure S1B), a finding consistent with previous report [49] and also with the HHcy condition. Similar experiments using B35 and Neuro-2a cells, a rat and a mouse origin neuroblastoma cells respectively, showed similar levels of cell death (40–50%) upon 5 mM Hcy treatment (Figure S1 C & D). We further checked Hcy toxicity in a more physiological set-up using cultured primary cortical neurons from rat. Primary neurons showed more sensitivity to Hcy and a similar level of cell death (almost 40%) was achieved by 0.75 mM Hcy treatment for 24 h (Figure S1E). To prove that the cell death observed was not simply due to amino acid toxicity and the specificity of Hcy, we treated neuroblastoma cells with similar doses of Met and Cys for 24 h. Both these amino acids associated with Hcy metabolism had a negligible effect on neuronal cell death (Figure S1F). To further test the tissue specific deleterious effect of Hcy, we treated HEK293 (Kidney), HeLa (Cervix), MCF7 (Breast) and A549 (Lung) cells with 5 mM Hcy for 24 h. In all these cells we found a negligible (˂10%) toxic effect of Hcy (Figure S1G). So, it indicates that Hcy has a species independent neuron specific toxicity.

Since apoptosis has been implicated in HHcy in a few previous studies [50, 51], we assessed the mode of cell death in our system. Annexin V/PI assay confirmed that Hcy-induced neuronal toxicity was mainly apoptotic (Fig. 1B and Figure S1H). We then determined whether Hcy-induced apoptosis was intrinsic in nature involving mitochondria as mitochondrial alteration was also reported in HHcy [52]. To test that we first measured mitochondrial membrane potential (ΔѰm) using potentiometric fluorescent dyes TMRE and DiOC6 in control and Hcy-treated cells. Hcy treatment showed 60–70% loss of ΔѰm compared to control cells (Fig. 1C and S1I). Next, we determined the outer mitochondrial membrane (OMM) permeabilization [53], a hallmark of mitochondrial apoptosis, in treated and untreated cells by employing two different methods. Firstly, we measured Cyto c release from mitochondria using a cyto c-GFP construct. Imaging at single cell level using Cyto c-GFP expressing primary neurons showed OMM permeabilization by Hcy as evident by loss of punctuate distribution of Cyto c in treated cells. Almost 60% of the Hcy treated neurons showed mitochondrial Cyto-c release (Fig. 1D). We further confirmed cyto c release from mitochondria in Hcy treated cells by immunoblot of separated mitochondrial and cytosolic fractions (Fig. 1E). As OMM permeabilization is frequently associated with loss of mitochondrial integrity, we tested mitochondrial morphology upon Hcy treatment. Mitochondrial fragmentation was confirmed in Hcy-treated neurons expressing a mitochondrial matrix targeted mtDsRed construct (Fig. 1F). Transmission electron microscopic studies of cellular sections further showed Hcy-induced loss of normal mitochondrial morphology as evident by a substantial presence of fragmented and swollen mitochondria with disrupted cristae (Fig. 1G). Next, we measured the activation of caspase-3 upon Hcy treatment to confirm the execution phase of intrinsic apoptosis. Our data confirmed that Hcy treatment activated caspase-3 in neuronal cells as evident by increased cleaved caspase-3 band in western blot and also the increased caspase-3 enzyme activity (Fig. 1H & I; S1J). Finally, we showed that neurotoxicity upon Hcy treatment was prevented by caspase-3 inhibitor Z-VAD-FMK (Fig. 1J and S1K). Taken together, the results demonstrate that Hcy-induced neurotoxicity is associated with mitochondrial dysfunction and intrinsic apoptosis.

### ER stress generated by Homocysteine causes mitochondrial dysfunction and neuronal toxicity

**Fig. 2:**
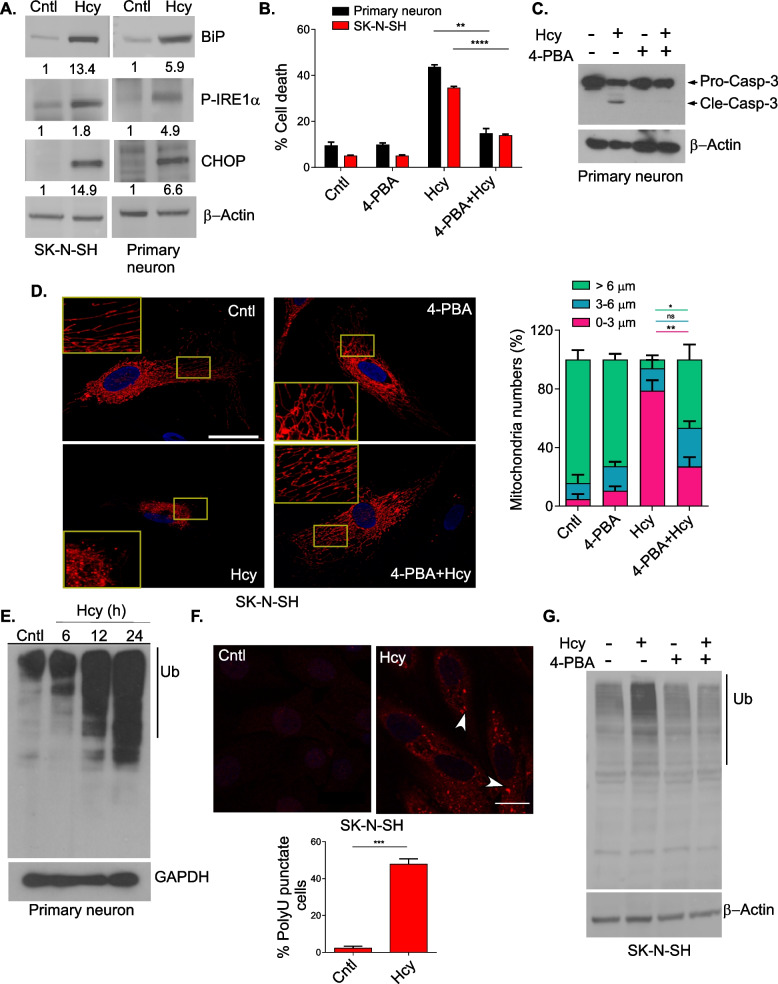
ER stress generated by Hcy causes mitochondrial dysfunction and neuronal toxicity. **A** Representative immunoblots showing expression levels of ER stress markers in Hcy treated SK-N-SH (left) and primary neurons (right). β-actin was used as a loading control. Densitometric values in the blots represent the ratio of respective protein signal to β-actin signal normalized to control. **B** Bar graphs showing rescue of Hcy-induced cell death in SK-N-SH and primary neurons by the chemical chaperone; 4-PBA. **C** Immunoblot analysis showing that only Hcy-condition causes caspase-3 activation (Cle-Casp-3) from the inactive pool of procaspase-3 (Pro-Casp-3) and the blockage in cleavage and activation of caspase-3 by 4-PBA in Hcy-treated primary neurons. Arrows marking the bands for respective inactive and active forms of Caspase-3. **D** Representative confocal images showing the rescue of mitochondrial fragmentation by 4-PBA in mitoDsRed expressing SK-N-SH cells upon Hcy treatment. Insets showing zoomed portions of the images and bar graph showing the average length distribution of different mitochondrial populations. Scale bar 20 µm. **E** Representative immunoblot showing time dependent increase in polyubiquitinated protein load in Hcy-treated primary neurons. GAPDH was used as a loading control. **F** Visualization of the increase in polyubiquitinated protein puncta by immunocytochemistry in Hcy-treated SK-N-SH cells (upper panel). Confocal images with scale bar 20 µm. Bar diagram (lower panel) showing the percent count of puncta containing cells. **G** Representative immunoblot showing the rescue of polyubiquitinated protein load by 4-PBA in Hcy treated SK-N-SH cells. β-actin was used as a loading control. Concentration of Hcy was 5 mM for SK-N-SH cells and 0.75 mM for primary neurons. 1 mM of 4-PBA was used. Data are shown as mean ± SEM with *n* ≥ 3. **P < 0.01.***P < 0.001.****P < 0.0001

Several previous reports mentioned that Hcy generates ER stress in different cellular systems [16, 54] and increased ER stress markers were also reported in fibroblasts derived from severe HHcy patients [55]. However, it is not clear whether ER stress solely causes the deleterious effect of Hcy in cells or whether there is some crosstalk between ER and mitochondria which finally culminates to cellular demise. To investigate it, we first tested the effect of Hcy on ER stress in our experimental set-up. Hcy treatment resulted in ER stress in both primary neurons and SK-N-SH cells (Fig. 2A), as evident by the significant induction of ER chaperones GRP78 (also named BiP), ER stress associated apoptotic marker CHOP (C/EBP homologous protein) and phosphorylation of IRE1 (inositol-requiring enzyme-1), three well-known ER stress indicators. We next evaluated whether this ER stress was necessary for the deleterious effect of Hcy on neuronal cells. For this purpose we tested 4-phenylbutyric acid (4-PBA) and Tauroursodeoxycholic acid (TUDCA), two chemical chaperones with known ER stress reducing activity in cellular and animal models [56], in our system. We first confirmed that Hcy-induced ER stress markers were rescued upon the treatment of the cells by 1 mM of 4-PBA (Figure S2A). Moreover, 4-PBA significantly reduced Hcy-induced cell death in both primary neurons and SK-N-SH cells (Fig. 2B) and also in B35 rat neuroblastoma cells (Figure S2B). Treatment of cells with TUDCA also showed similar results against Hcy-induced neuronal death (Figure S2C). This depicts that ER stress generated by Hcy is causal to its toxic effect on neuronal cells. Next, we investigated whether Hcy-induced ER stress caused mitochondrial dysfunction and apoptosis in neuronal cells. Indeed, we saw the rescue of mitochondrial fragmentation (Fig. 2D and Figure S2D) and caspase-3 activation (Fig. 2C) by 4-PBA against Hcy treatment which could explain the rescue of Hcy-induced neuronal death by 4-PBA.

ER stress frequently crosstalks with mitochondria to exert its deleterious effect mainly through ROS generation, proteotoxicity and ER calcium release and consequent mitochondrial calcium overload [57]. Next, we studied the preferred pathway of ER mitochondria crosstalk in Hcy-induced neurotoxicity. First, we measured ROS generation in neuronal cells by using two different fluorescent dyes H2DCFDA and MitoSOX Red upon Hcy treatment and found no significant change in ROS level in both primary cortical neurons and SK-N-SH cells (Figure S2 E & F). Moreover, N-acetyl cysteine (NAC), one of the most commonly used ROS scavengers had no protective effect against Hcy toxicity (Figure S2G). Next, we measured the level of mitochondrial matrix calcium upon Hcy treatment using a mitochondrial matrix targeted fluorescence dye Rhod-2-AM. Our result showed no significant change in mitochondrial calcium level in Hcy treated cells compared to the control (Figure S2H). We further negated the role of calcium-induced mitochondrial apoptosis by Hcy using mitochondrial calcium uptake blocker Ruthenium Red (RuRed) and mitochondrial permeability transition pore (PTP) blocker Cyclosporine-A (CsA), widely used as blockers of calcium induced mitochondrial apoptosis [58]. Our data confirmed that both RuRed and CsA did not protect neuronal cells against Hcy toxicity (Figure S2 I & S2 J). Finally we measured the level of proteotoxic stress in neuronal cells upon Hcy treatment. We saw the increase of Hcy-induced proteotoxic stress as evidenced by the increased polyubiqutinated protein load in a time dependent manner (Fig. 2E). Moreover, using immunocytochemistry we further visualized the increased presence of toxic protein aggregates in HHcy condition compared to the untreated controls (Fig. 2F). Next, we checked whether protein aggregates generated by Hcy-induced ER stress were indeed the cause of cellular demise. To prove it, we treated the neuronal cells with Hcy in presence of the ER stress blocker 4-PBA. As expected, 4-PBA rescued the Hcy-induced polyubiquitinated protein load as evidenced by immunoblot analysis (Fig. 2G) and also the number of aggregated punctae visualized through immunocytochemistry (Figure S2K). In summary, the above results indicate that Hcy-induced ER stress is an upstream event which causes mitochondrial dysfunction and apoptosis in neuronal cells and the crosstalk between ER and mitochondria is mainly dependent on proteotoxicity generated by increased aggregated proteins upon Hcy treatment.

### Elevated Hcy downregulates basal autophagy in neuronal cells

**Fig. 3:**
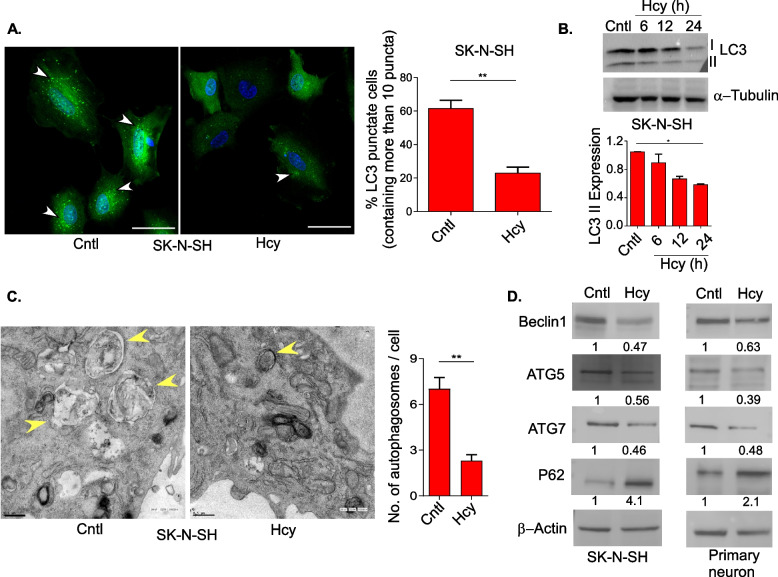
Elevated Hcyo downregulates basal autophagy in neuronal cells. **A** Left panel: representative confocal images showing LC3-GFP puncta in control and Hcy-treated SK-N-SH cells transiently transfected with LC3-GFP construct. Arrowheads represent GFP-LC3 puncta. Scale bar 20 µm. Right panel: percent quantitation of LC3-GFP puncta containing cells (≥ 10 puncta per cell). **B** Representative immunoblot (upper panel) and densitometric quantitation (lower panel) showing a time dependent decrease in lipidated LC3-II form (marker of autophagy) in Hcy-treated cells. β-tubulin was used as a loading control. **C** Representative TEM images showing the decrease in autophagic vesicles upon Hcy treatment in SK-N-SH cells. Arrowheads represent double membrane autophagic vesicles. Scale bar 0.5 µm. Bar graph showing the quantitation of the TEM images representing the number of autophagic vesicles per cell. **D** Representative immunoblots showing a decreased expression of major autophagic proteins (Beclin1, ATG5, ATG7) and a concomitant increase in cargo protein (P62) in SK-N-SH (left) and primary neurons (right), confirming autophagic suppression by Hcy. β-actin was used as a loading control. Densitometric values in the blots represent the ratio of respective protein signal to β-actin signal normalized to control. Concentration of Hcy was 5 mM for SK-N-SH cells and 0.75 mM for primary neurons. Data are shown as mean ± SEM with *n* ≥ 3. *P < 0.05. **P < 0.01

Next, we studied the effect of HHcy on autophagic process in neuronal cells as induction of autophagy is a well-established protective mechanism against ER stress [35, 59]. Moreover, maintenance of basal level autophagy is utmost important for neuronal homeostasis and health [60]. Firstly, the LC3-GFP expressing neuronal cells showed less LC3-positive punctate structures upon Hcy treatment (Fig. 3A and S3A) indicating suppression of autophagy. We also measured the level of LC3-I to LC3-II conversion by immunoblotting which showed a steady decrease of LC3-II form in Hcy treated cells (Fig. 3B and Figure S3B). To check whether Hcy induced autophagic dysregulation in our system was mTOR dependent or not, we measured the levels of total and phosphorylated mTOR and its downstream substrates P70:S6K and 4E-BP1 by immunoblotting. Our data showed that indeed Hcy treated cells had higher levels of phosphorylated mTOR and its targets (Figure S3C) which support a couple of previous reports that Hcy activates mTOR [40, 61]. To further confirm the suppression of autophagy by Hcy we did electron microscopy of control and treated cells and found a negligible presence of double-membraned autophagosome in the treated groups (Fig. 3C). In addition, we also measured the level of key autophagy related proteins like Beclin1, ATG5 and ATG7 and all these three proteins showed significant decrease in Hcy-treated neuronal cells (Fig. 3D) that further indicated the lowering of autophagy in HHcy. Finally, we measured the level of P62 in control and Hcy-treated cells. P62 is a LC3 interacting cargo protein for helping selective autophagic degradation and thus the total cellular levels of P62 inversely correlate with autophagic activity [62]. Unlike the lowering of LC3-II forms, P62 levels were highly increased upon Hcy treatment (Fig. 3D), further suggesting the suppression of autophagy in Hcy-treated neuronal cells. Collectively all these results indicate that the neuronal autophagy is downregulated in HHcy.

### Autophagic suppression by Hcy is the cause of ER stress in Hyperhomocysteinemic neurons

**Fig. 4:**
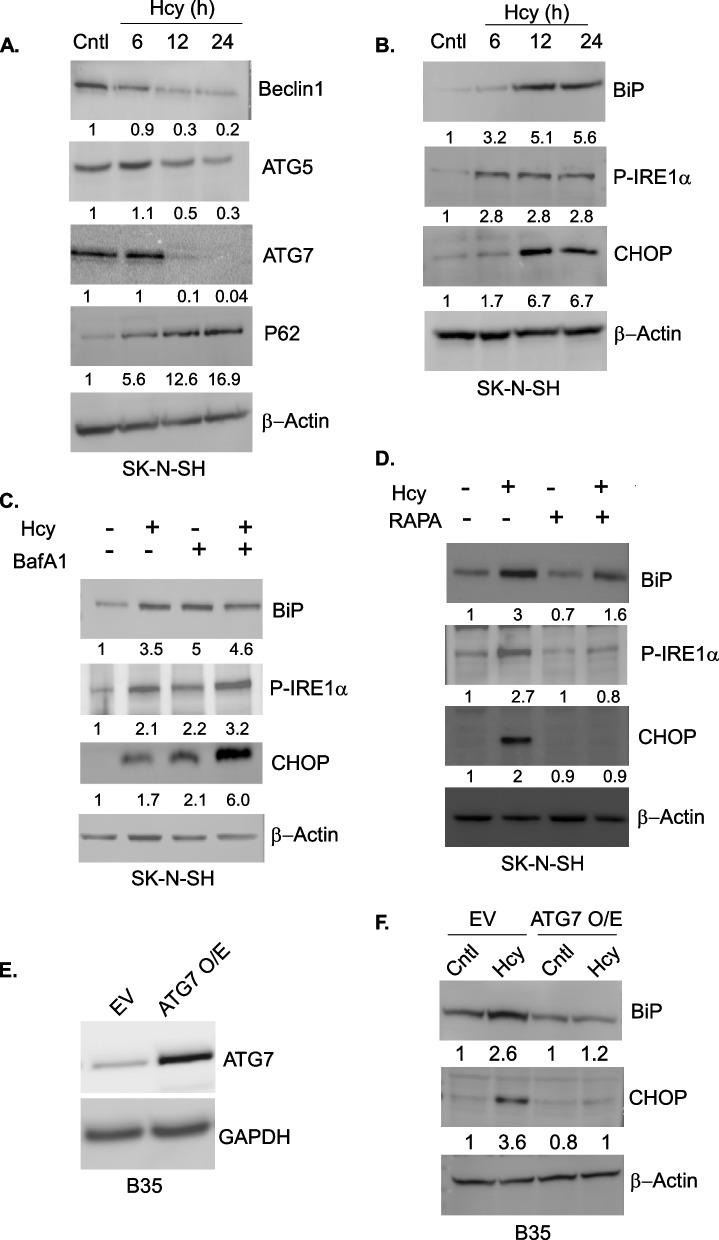
Autophagic suppression by Hcy is the cause of ER stress in Hyperhomocysteinemic neurons. Representative immunoblots of same cell lysates showing a time dependent suppression of autophagy (**A**) and an activation of ER stress (**B**) by Hcy in primary neurons. β-actin was used as a loading control. Densitometric values in the blots represent the ratio of respective protein signal to β-actin signal normalized to control. Representative immunoblots of ER-stress marker proteins showing increased expression by autophagic inhibitor BafA1 (20 nM) treatment (**C**) and decreased expression by autophagic activator RAPA (200 nM) treatment (**D**) in Hcy-treated SK-N-SH cells. β-actin was used as a loading control. Densitometric values in the blots represent the ratio of respective protein signal to β-actin signal normalized to untreated control. **E** Immunoblot confirming higher expression of ATG7 protein in a stable neuroblastoma cell line overexpressing ATG7. GAPDH was used as a loading control. **F** Representative immunoblots showing decreased expression of ER-stress markers after Hcy-treatment in ATG7 overexpressing (constitutively activated autophagy) B35 cells compared to vector control cells. β-actin was used as a loading control. Densitometric values in the blots represent the ratio of respective protein signal to β-actin signal normalized to control. Concentration of Hcy was 5 mM. Data are shown as mean ± SEM with *n* ≥ 3

Though we could establish Hcy-induced ER stress as the cause of toxicity in the neuronal cells, the origin of this stress is not clear at molecular level. It has also been shown that defective hepatic autophagy promotes ER stress in obese mice [63] and constitutive pancreatitis and ER stress in pancreas specific ATG7 [64] and ATG5 [65] mice. As we found a low level of autophagy in Hcy-treated neuronal cells, we wondered whether this lowering of autophagic process could be the reason for Hcy-induced ER stress. To test this possibility, we studied the effect of Hcy on autophagy and ER stress at cellular level in a time course experiment. Hcy treatment caused a time dependent decrease of autophagy in both neuronal cells and primary neurons as evident by the immunoblot analysis of Beclin1, ATG5 and ATG7 (Fig. 4A and Figure S4A). It was further confirmed by the concomitant increase at the level of P62 (Fig. 4A and Figure S4A). We analyzed the level of ER stress markers in similar samples by immunoblotting which showed a time dependent increase of known ER markers BiP, phosphorylated form of IRE1 (p-IRE1) and CHOP (Fig. 4B and Figure S4B). This indicates a direct correlation of autophagic downregulation and ER stress generation in HHcy cells. If lowering of autophagy is indeed the cause of ER stress then we expect that blocking of autophagy along with Hcy treatment should not change the ER stress significantly whereas activation of autophagy should have a protective role against Hcy-induced ER stress. To confirm it, we first treated the cells with the autophagic blocker Bafilomycin A1 (BafA1) along with Hcy. Immunoblot analysis showed that BafA1 alone was able to activate ER stress markers almost at the level of Hcy and maintained that level even in the presence of Hcy (Fig. 4C). Next, we tested the status of ER stress generated by Hcy in presence of Rapamycin (Rapa), a pharmacological activator of autophagy. Treatment with Rapa significantly rescued Hcy-induced ER stress in neuronal cells as evident from the immunoblot results of BiP, p-IRE1 and CHOP (Fig. 4D). As several pharmacological agents frequently have nonspecific effects on cells, we further confirmed the protective effect of Rapa in a cellular model of activated autophagy. For that purpose we generated a stable ATG7 overexpressing B35 neuroblastoma cells (Fig. 4E). Immunoblotting results pointed out that indeed ATG7 overexpressing cells were resistant to Hcy-induced ER stress (Fig. 4F). Finally, to confirm autophagic suppression in HHcy as a cause of the downstream ER stress generation in neuronal cells, we treated our cells with a known ER stress inducer Tunicamycin as a positive control. Unlike Hcy, Tunicamycin activated ER stress with a simultaneous increase in autophagy as evidenced by the immunoblot analysis of ER stress and autophagic marker proteins (Figure S4C). Taken together, these data demonstrate that Hcy-induced autophagic downregulation in neuronal cells is the probable cause of the ER stress in HHcy.

### Autophagic modulation changes Hcy-induced proteotoxicity and consequent neuronal cell death

**Fig. 5:**
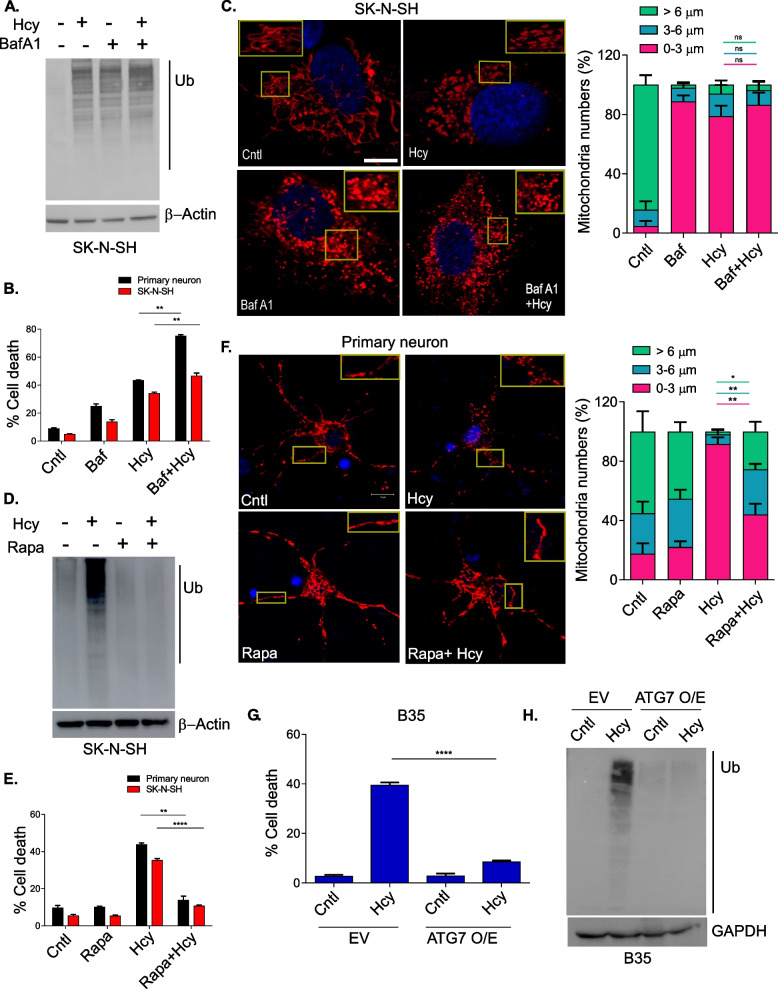
Autophagic modulation changes Hcy-induced proteotoxicity and consequent neuronal cell death. **A** Representative immunoblot showing autophagic blocker BafA1 (20 nM) has equal or additive effect on polyubiquitinated protein load in Hcy alone or combined treatment in SK-N-SH cells. β-actin was used as a loading control. **B** Bars showing increased percent cell death by BafA1 in Hcy-treated neuronal cells. **C** Representative confocal images confirming increased mitochondrial fragmentation by BafA1 in mitoDsRed expressing SK-N-SH cells upon Hcy treatment. Insets showing zoomed portions of the images and bar graph showing the average length distribution of different mitochondrial population. Scale bar 10 µm. Pharmacological activation of autophagy by RAPA (200 nM) showing rescue of polyubiquitinated protein load in immunoblots (**D**) and consequent protection against percent cell death (**E**) in neuronal cells upon Hcy treatment. β-actin was used as a loading control in immunoblot analysis. **F** Representative confocal images showing the rescue of mitochondrial fragmentation by RAPA in Hcy treated primary neurons. Insets showing zoomed portions of the images and bar graph showing the average length distribution of different mitochondrial populations. Scale bar 10 µm. Genetic activation of autophagy showing rescue of percent cell death (**G**) and ubiquitinated protein load (**H**) upon Hcy treatment in B35 neuroblastoma cells stably overexpressing ATG7. GAPDH was used as a loading control in immunoblot analysis. Concentration of Hcy was 5 mM for SK-N-SH cells and 0.75 mM for primary neurons. Data are shown as mean ± SEM with *n* ≥ 3. **P < 0.01. ****P < 0.0001

We have already shown above that Hcy-induced neuronal cell death is associated with sustained ER stress and proteotoxicity. Subsequently, we have also established that Hcy-induced suppression of autophagy is the main cause for this sustained ER stress. So, it is rational to predict that further suppression of upstream autophagy should have a negative effect on proteotoxicity and consequent cell death upon Hcy treatment whereas activation of autophagy should protect these deleterious effects in similar conditions. To investigate it we measured total polyubiquitinated protein load and cell death upon Hcy treatment in presence of BafA1. Our results showed that there was an increase in toxic protein load (Fig. 5A), neuronal cell death (Fig. 5B) and mitochondrial fragmentation (Fig. 5C) in combination of BafA1 and Hcy compared to the only Hcy treated groups. We saw similar trends in terms of cell death using an early stage autophagy blocker Wortmannin (Wmn) like that of the late stage autophagy blocker BafA1 (Figure S5A). Next, we studied the effect of autophagic activation on Hcy-induced proteotoxicity and neuronal cell death. We first tested it pharmacologically by using Rapa. Unlike BafA1, Rapa treatment rescued the total polyubiqutinated protein load (Fig. 5D and Figure S5B) as well as the deleterious effect of Hcy (Fig. 5E and Figure S5C) and mitochondrial structure (Fig. 5F) in neuronal cells. To further confirm the protective effect of autophagic activation on Hcy toxicity, we used the genetic model of ATG7 overexpressing neuronal cells. Our results suggested that the ATG7 overexpressed cells indeed had significantly low amounts of toxic protein load (Fig. 5H) and concomitant cell death (Fig. 5G) compared to the vector expressing control cells upon Hcy treatment. To confirm further that these effects of ATG7 were not a gene specific effect rather a generalized effect of autophagy activation, we performed similar experiments by activating autophagy using another autophagy related gene ATG5. We saw similar suppression in terms of total polyubiquitinated protein load and cell death upon Hcy treatment in ATG5 overexpressing neuronal cells (Figure S5D, E & F). In summary, these results indicate that autophagic suppression is indeed the cause of sustained ER stress in HHcy and activation of upstream autophagy would be enough to nullify the ER stress and concomitant neuronal toxicity by Hcy.

### Hyperhomocysteinemic *Drosophila melanogaster* shows downregulation of the autophagy genes and upregulation of the stress response genes at systemic level

**Fig. 6:**
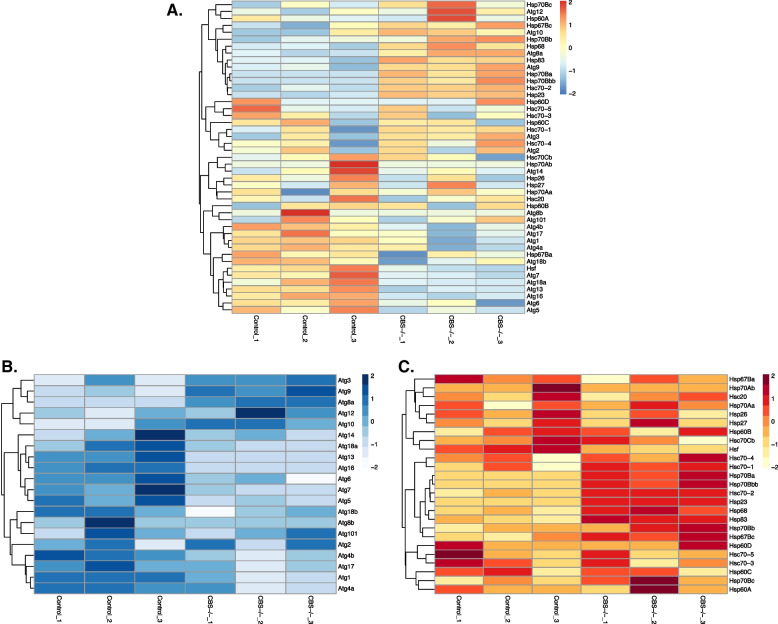
Hyperhomocysteinemic *Drosophila melanogaster* shows downregulation of the autophagy genes and upregulation of the stress response genes at systemic level. **A** Heat map illustrating RNA-Seq differential expression data for autophagy and stress response pathway genes in control versus CBS knockout (whole body) flies. Pairwise comparisons with wild type are shown. Red positive log fold change (log2FC). Blue negative log2FC. Heat map showing pathway specific expression of autophagy (**B**) and stress response (**C**) genes in the same knockout flies. Pairwise comparisons with wild type control strains are shown. Respective color legends representing log fold changes (log2FC). Three sets of data from 3 different wild type control and knockout flies (CBS-/-) were used for analysis.

Cystathionine beta synthase (CBS) is the first and rate limiting enzyme in the transsulfuration pathway that synthesizes cysteine from Hcy. Mutation or deficiency in CBS is associated with severe homocysteinemia at systemic level [66]. In a recent work Zatsepina et al. reported the generation and genome-wide transcriptional profiling of transsulfuration genes in *Drosophila melanogaster* [67]. We downloaded and reanalyzed the raw read counts from the deposited sequence data (GSE148109, NCBI GEO database) of whole body CBS knockout (CBS-/-) flies to check the differential expression of the genes associated with autophagy and stress response pathways. As the CBS-/- flies were viable and fertile, we hypothesized from our cellular data that any systemic changes in autophagy could well be reflected in the stress response pathways for maintaining homeostasis. Indeed we saw an overall differential gene expression pattern in autophagy and heat shock related genes in control and CBS-/- flies comparing three independent subjects for each condition as represented in the expression heat map (Fig. 6A). To further assess the changes in the specific components of these pathways in control and knockout flies we compared the gene expression patterns of the autophagy related genes (Atg) and heat shock response genes (Hsp/Hsc). Our analysis showed that almost 70% of the Atg genes (14 out of 20) were downregulated (Fig. 6B) and concomitantly almost 60% of the heat shock genes (14 out of 25) were upregulated (Fig. 6C) in CBS-/- flies compared to the control flies. Overall, the gene expression analysis data confirm that HHcy indeed downregulate autophagy at systemic level and also indicate that the resulting altered proteostasis due to autophagic alteration may be balanced by a systemic upregulation of the stress response pathway for the survival and the fertility of the knockout flies.

### Hyperhomocysteinemic zebrafish develops brain damage associated with reduced autophagy, constitutive ER stress and proteotoxicity

**Fig. 7:**
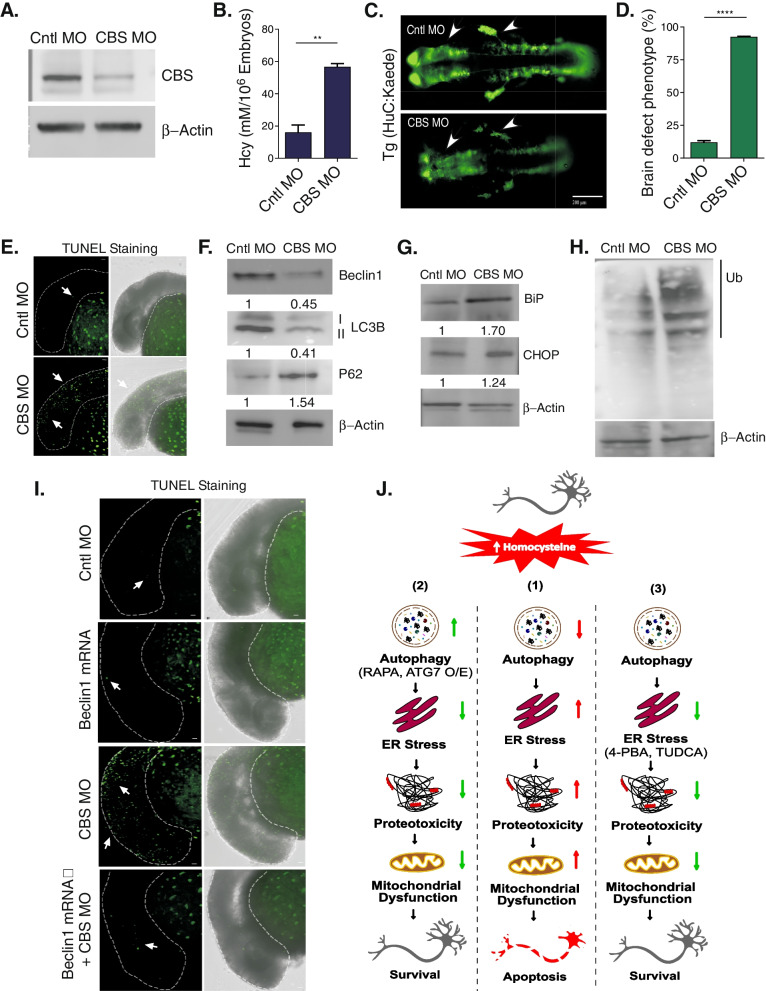
Hyperhomocysteinemic zebrafish develops brain damage associated with reduced autophagy, constitutive ER stress and ubiquitinated protein load. **A** Immunoblot of zebrafish embryos injected with Cntl MO or CBS MO at 24 hpf. confirminging downregulation of CBS. β-actin was used as a loading control. **B** Concentration of Hcy (mM) at 24 hpf in Cntl MO and CBS MO zebrafish embryos as measured by HPLC. CBS morphants showing HHcy. **C** Representative confocal images (10X) showing brain defects in a brain specific Kaede (green) expressing transgenic fish Tg(HuC:Kaede) embryos injected with Control MO or CBS MO. Arrows showing neuronal damage in CBS morphants. Scale bar 200 µm. **D** Bar diagram represents percentage of embryos with brain defect in the CBS MO compared to Cntl MO. More than 200 embryos were counted phenotypically. **E** Representative images (10X) of TUNEL staining in the brain of Cntl MO and CBS MO embryos. Left panel showing fluorescent images only and right panel showing bright field and fluorescent image overlap. Arrowheads indicate excess number of apoptotic cells in the head region of CBS morphants. Scale bar 25 µm. The area highlighted by the dotted lines indicating the brain region of the embryos. Representative western blots showing markers for intrinsic suppression of autophagy (**F**) and increase in ER stress markers (**G**) as well as polyubiquitinated protein load (**H**) in CBS morphants compared to controls. β-actin was used as a loading control. Densitometric values in the blots represent the ratio of respective protein signal to β-actin signal normalized to control. **I** Representative confocal images showing rescue of apoptotic cell death in the brain region of CBS morphant upon autophagic activation by overexpressing Beclin1. Left panel showing fluorescent images only and right panel showing bright field and fluorescent image overlap. Scale bar 20 µm. Arrowheads indicate attenuation of excess apoptosis in Beclin1 mRNA and CBS MO co-injected embryos as compared to only CBS MO injected ones. The area highlighted by the dotted lines indicating the brain region of the embryos. **J** Schematic showing pathways of Hcy induced neurotoxicity (1). Rescue of neurotoxicity either by autophagic activation upstream (2) or by blocking ER stress downstream (3). Data are shown as mean ± SEM with *n* ≥ 3. **P < 0.01. ****P < 0.0001

To examine the impact of elevated Hcy in a live animal model, we used a CBS knockdown approach to generate a hyperhomocysteinemic zebrafish and used it to study the effects of Hcy on neuronal homeostasis in the developing brain of zebrafish embryos. CBS gene is duplicated in zebrafish as compared to a single copy in mammals and this type of duplication of several different genes in zebrafish are well characterized [68]. To generate a hyperhomocysteinemic zebrafish, gene specific antisense morpholinos (MO) targeted to the ATG regions (CBSa and CBSb MOs from Gene-Tools) were injected into embryos at 1–2 cell stage for translational knockdown. A mismatched MO (Gene-Tools) was used as a negative control. First, we confirmed the efficacy of MOs in targeting and blocking protein translation by immunoblotting and indeed 24 h post fertilization (hpf) morphant embryos showed significant downregulation of CBS (Fig. 7A). We also confirmed that almost 85% of control and CBS morphant embryos survived compared to the non-injected control embryos (Figure S6A). To confirm whether CBS morphants were indeed hyperhomocysteinemic, we measured Hcy level by HPLC in control and CBS morphant embryos. Our data showed that there was almost a fivefold increase in Hcy level in CBS morphant embryos (Fig. 7B) that nicely mimics our result of intra-cellular level of Hcy upon treatment (Figure S1). Then we compared the morphology of 24 hpf CBS morphant embryos with that of control morphant and non-injected control embryos. Though no gross phenotypic defects were apparent, a careful observation showed clear abnormalities in head and CNS development in HHcy zebrafish embryos at 24 hpf with a not so well-defined brain structure (Figure S6B). To study the brain abnormality further in HHcy, we used a transgenic zebrafish line Tg (HuC:Kaede), expressing the fluorescent protein Kaede specifically in neurons under the control of HuC promoter [45]. In vivo morphological analysis showed that like wild type HHcy morphants, transgenic CBS morphant embryos also had similar type of abnormal head development associated with disorganization of CNS structure (Fig. 7C). Higher magnification analyses on CBS morphant Tg (HuC:Kaede) at 24 hpf confirmed the loss of neurons as evident by less Kaede signal as well as the morphological abnormality of anterior CNS structure compared to the control morphant transgenic embryos (Fig. 7C). Almost 80% of the CBS morphant embryos showed this abnormal brain phenotype (Fig. 7D). As in our cellular models we already established the link between HHcy and neuronal apoptosis, we next studied whether apoptosis was associated with the brain defects observed in HHcy zebrafish embryos. For this purpose whole-mount TUNEL-staining was used to detect apoptotic cells in control and CBS morphant embryos analyzed at 24 hpf. Minimal evidence of apoptosis was found in control morphant, whereas a highly increased number of TUNEL-positive cells were detectable in the head region of CBS morphant embryos, suggesting that the developmental defects of CNS was indeed associated with an increase of neuronal apoptosis (Fig. 7E). Next we investigated the status of autophagy, ER stress and toxic protein load in control and CBS morphant zebrafish embryos as our cellular model data showed that HHcy caused neuronal apoptosis through autophagic downregulation and concomitant high ER stress and proteotoxicity. In order to elucidate the status of autophagy in HHcy zebrafish, LC3-II level was compared in CBS morphant and control morphant embryos at 1 days post fertilization (dpf) using immunoblotting. Indeed CBS morphant embryos showed less LC3-II levels compared to the control embryos which indicated lowering of autophagy in HHcy zebrafish (Fig. 7F). Suppression of basal autophagy in HHcy at in vivo level was further confirmed by lowering of Beclin1 and concomitant increase of P62 level in CBS MO injected embryos compared to the respective controls (Fig. 7F). According to our cellular data, we expected that the lowering of basal autophagy in HHcy zebrafish should be associated with increased ER stress and toxic protein load. As expected, CBS morphant embryos showed constitutively higher ER stress than the control morphant embryos as evidenced by the high levels of ER stress markers BiP and CHOP in immunoblot analysis (Fig. 7G). Then we checked the level of total polyubiquitinated protein load as proteotoxic marker in control and HHcy zebrafish embryos. Immunoblot analysis confirmed a significant increase in total polyubiquitinated protein level in CBS morphant embryos compared to the control embryos (Fig. 7H). Finally, to affirm that the signaling axis described by us is indeed the reason for the neuronal apoptosis in CBS morphant embryos, we did an in vivo rescue experiment by activating upstream autophagy using Beclin1. Whole-mount TUNEL-staining showed that the number of TUNEL-positive cells were much less in the head region of the CBS morphant embryos co-injected with human Beclin1 mRNA compared to the only CBS morphant embryos (Fig. 7I). So, collectively our cellular and in vivo zebrafish data suggest the model where HHcy causes brain damage through mitochondrial apoptosis in neuronal cells (Fig. 7J). Upstream autophagic suppression by high Hcy causes the increased proteotoxic load and resultant constitutive ER stress that culminates to mitochondrial dysfunction and neuronal apoptosis.

## Discussion

Homocysteinuria, the accumulation of very high level of plasma Hcy, is classically designated as an inborn error of metabolism due to CBS deficiency and has a clinical prevalence of around 1/200000 births worldwide. Though it is generally considered as a rare genetic disorder, its prevalence is much higher (1/1800 births) in some populations and strongly linked to complications like intellectual disability, behavioral disorders and early onset stroke [69, 70]. Beyond that HHcy due to nutritional deficiency and aging has also been linked strongly with several neurological and neurodegenerative disorders [71]. Clinical trials on lowering of plasma Hcy level did not show a clearly measurable positive clinical impact on the development of neural pathologies [1] which indicates the possibility of higher Hcy dependent subtle damages of the brain at organ level. Therefore, it is necessary to elucidate the molecular pathways involved in Hcy-induced neurotoxicity for future tackling of cognitive and neurological manifestations during HHcy.

Here, we comprehensively describe the stepwise signaling cascades and its cause and effect correlation in Hcy-induced neurotoxicity. Our data suggest that neuronal cells are more vulnerable to Hcy-induced intrinsic apoptosis associated with mitochondrial damage, Cyto c release and caspase-3 activation and thus caspase-3 inhibitors rescue this toxic insult (Fig. 1 & S1). We have also identified that ER stress is the upstream event that triggers the Hcy-induced mitochondrial apoptosis, as blocking it by two different chemical chaperones rescue both the mitochondrial integrity and apoptosis (Fig. 2 & S2). Though several previous reports indicate the role of ER stress in Hcy-induced neurotoxicity but how it causes mitochondrial apoptosis is not clear. Conflicting reports exist on the role of oxidative damage and Ca^2+^ overload [23, 28, 29, 72] whereas the role of terminal UPR is not well defined in this process. In this work we showed no significant change in ROS and Ca^2+^ levels whereas an increase in ubiquitinated protein load in Hcy treated cells (Fig. 2 & S2). ROS scavengers, blockers of PTP and mitochondrial Ca^2+^ uniporter had no rescuing effect rather increased the apoptosis whereas controlling ubiquitinated protein load showed protection against it (Fig. 2 & S2). Thus, we established that the terminal UPR activation by toxic protein load in HHcy culminates in mitochondrial damage and apoptosis as previously reported in cases of unresolved ER stress [73].

In normal conditions the cellular response of misfolded protein stress is the activation of intrinsic quality control pathways to maintain homeostasis and survival [74]. However, being post-mitotic in nature, neurons are particularly dependent on autophagy and it is constitutively active in neurons as a part of their robust quality control mechanisms to support their long-term functionality and survival [75, 76]. In this line our data also showed an increase in autophagy markers upon tunicamycin-induced ER stress in neuronal cells (Figure S4). However, we saw autophagic suppression in neurons upon HHcy and this effect was associated with aberrant activation of the mTOR pathway (Fig. 3 & S3). This observation is supporting a previous report where hyperhomocysteinemic mice brain showed toxic build-up of β-amyloid and phospho-Tau due to autophagic downregulation that leads to AD-like neurodegeneration [40], though several other reports indicated autophagic activation and neural damage in HHcy [41, 77]. This apparent dichotomy may be explained as these later works either used ischemic models of brain injury or specific knockout mouse models and in line with the previous observation of Atg-deletion dependent prevention of severe neural damage after ischemic brain injury in mice [78].

Our observation on Hcy-induced sustained ER stress and suppression of autophagy in neuronal cells indicated the possibility of a cause and consequence relationship between these two processes. Our time-course analysis data showed a steady decrease in autophagic markers along with a concomitant steady increase in ER stress markers by Hcy (Fig. 4 & S4). Expectedly, pharmacological blocking of autophagy further increased the ER stress markers whereas pharmacological and genetic activation of autophagy mitigated the ER stress in neurons upon HHcy (Fig. 4). Consequently pharmacological blockers of autophagy further increased whereas pharmacological and genetic activator of autophagy rescued the overall ubiquitinated protein load, mitochondrial integrity and apoptosis by Hcy (Fig. 5 & S5). So, upstream autophagic suppression by Hcy triggered ER stress through the inefficient clearance of unfolded proteins, creating a maladaptive feed-forward mechanism that amplified ER stress further and mitochondrial apoptosis. This observation is also in support of previous reports on pancreatitis [64, 65] and on obesity-induced insulin resistance [63] where similar signaling cascade at cellular level causes respective pathologies.

At organismal level, CBS knockout mouse models of severe HHcy have been generated previously and homozygous animals showed neonatal lethality where almost 90% cbs-/- animals died by 2–4 weeks after their birth [79, 80]. Though liver failure and vascular complications were attributed to this neonatal death, the precise mechanisms associated with these organ damages are not known [66]. Very recently by using CRISPR/Cas9 approach, a homozygous CBS KO flies has been generated which are viable and fertile [67]. Our pathway-based comparison of the transcriptomic data from these WT and cbs-/- flies clearly showed that a systemic suppression of autophagic genes were counterbalanced by upregulation of stress response pathway genes (Fig. 6) which may well be the reason of their possible viability unlike cbs-/- KO mice. It also indicates that our cellular data on autophagic suppression as the cause of ER stress and apoptosis may be a generalized phenomenon of associated pathologies in HHcy.

In recent years several literature on humans and rodents describe the neural damage and compromised cognitive outcome in the offspring due to maternal HHcy [13, 14, 81, 82] though the molecular pathways in this regard are not well established. Our data on the HHcy zebrafish model also support these basic results as both neuronal apoptosis and developmental defects in the brain were observed (Fig. 7). Our data in HHcy fish also indicate the suppression of autophagy, increased toxic protein load and ER stress constitutively at systemic level that further endorsed our cellular data on Hcy-induced toxicity (Fig. 7). Most importantly, we were able to rescue the neuronal apoptosis in our HHcy zebrafish model by activating upstream autophagy and that further confirmed the role of the signaling axis proposed by us in Hcy-induced neurotoxicity (Fig. 7). Of note, we purposefully used a knockdown approach over a KO model in fish to establish the role of these pathways in brain damage for overcoming the intrinsic compensation of these interlinked pathways of homeostasis, which we saw in the KO flies. It also supports that our proposed mechanism of Hcy-induced neurotoxicity is playing a central role in early brain aberrations reported during development in an Hcy-rich environment. It is worth mentioning here that in line with our observation, a new drug combination of 4-PBA and TUDCA showed good therapeutic efficacy against ALS and it is waiting for the final FDA approval for treatment [83]. However, future work on the precise mechanism by which Hcy activates mTOR and consequently downregulates autophagy will be an interesting area to explore. A few recent studies reported the transgenerational effect of Hcy in brain pathologies [84, 85] and that opens up the possibilities of some epigenetic modifications in the brain during HHcy. In the backdrop of this present work, it will be interesting to study further to check whether epigenetic suppression of the autophagic pathway could be an early step and might be a reason for the neuronal damage reported widely in HHcy.

In conclusion, the present work informs on the role of crosstalk between autophagy, ER stress, mitochondrial dysfunction and apoptosis as a cause and consequence manner in Hcy-induced neurotoxicity. It also delineates the sequential perturbation of the fine-balance in autophagy-ER stress-mitochondrial apoptosis triad as a central mechanism to the brain pathologies associated with HHcy. Rescue of neuronal damage either by autophagic activation upstream or by blocking ER stress downstream in this report will help further to device new strategies for the treatment of neural and cognitive pathologies reported in HHcy, as the present therapy of vitamin and folate supplementation is not entirely successful to alleviate these problems.

### Supplementary Information

**Additional file 1.**

## Data Availability

The transcriptome dataset analyzed in the current study has been previously published in Zatsepina et al. (2020) [62] and is available at the NCBI Gene Expression Omnibus (accession no. GSE148109), https://www.ncbi.nlm.nih.gov/geo/query/acc.cgi?acc.

## References

[1] Hannibal L, Blom HJ (2017). Homocysteine and disease: causal associations or epiphenomenons?. Mol Aspects Med.

[2] Zaric BL, Obradovic M, Bajic V, Haidara MA, Jovanovic M, Isenovic ER (2019). Homocysteine and hyperhomocysteinaemia. Curr Med Chem.

[3] Troen AM (2005). The central nervous system in animal models of hyperhomocysteinemia. Prog Neuropsychopharmacol Biol Psychiatry.

[4] Luzzi S, Papiri G, Viticchi G, Baldinelli S, Fiori C, Silvestrini M (2021). Association between homocysteine levels and cognitive profile in Alzheimer’s disease. J Clin Neurosci.

[5] Kalecký K, Ashcraft P, Bottiglieri T. One-carbon metabolism in Alzheimer’s disease and Parkinson's disease brain tissue. Nutrients. 2022;14. 10.3390/nu14030599. 10.3390/nu14030599 PMC8838558 35276958

[6] Fan X, Zhang L, Li H, Chen G, Qi G, Ma X (2020). Role of homocysteine in the development and progression of Parkinson’s disease. Ann Clin Transl Neurol.

[7] Oliveira SR, Flauzino T, Sabino BS, Kallaur AP, Alfieri DF, Kaimen-Maciel DR (2018). Elevated plasma homocysteine levels are associated with disability progression in patients with multiple sclerosis. Metab Brain Dis.

[8] Farkas SA, Böttiger AK, Isaksson HS, Finnell RH, Ren A, Nilsson TK (2013). Epigenetic alterations in folate transport genes in placental tissue from fetuses with neural tube defects and in leukocytes from subjects with hyperhomocysteinemia. Epigenetics.

[9] Bottiglieri T, Laundy M, Crellin R, Toone BK, Carney MW, Reynolds EH (2000). Homocysteine, folate, methylation, and monoamine metabolism in depression. J Neurol Neurosurg Psychiatry.

[10] Casas JP, Bautista LE, Smeeth L, Sharma P, Hingorani AD (2005). Homocysteine and stroke: evidence on a causal link from mendelian randomisation. Lancet.

[11] Martínez-Vega R, Garrido F, Partearroyo T, Cediel R, Zeisel SH, Martínez-Álvarez C (2015). Folic acid deficiency induces premature hearing loss through mechanisms involving cochlear oxidative stress and impairment of homocysteine metabolism. FASEB J.

[12] Navneet S, Zhao J, Wang J, Mysona B, Barwick S, Ammal Kaidery N (2019). Hyperhomocysteinemia-induced death of retinal ganglion cells: the role of Müller glial cells and NRF2. Redox Biol.

[13] Blaise SA, Nédélec E, Schroeder H, Alberto J-M, Bossenmeyer-Pourié C, Guéant J-L (2007). Gestational vitamin B deficiency leads to homocysteine-associated brain apoptosis and alters neurobehavioral development in rats. Am J Pathol.

[14] Shcherbitskaia AD, Vasilev DS, Milyutina YP, Tumanova NL, Zalozniaia IV, Kerkeshko GO (2020). Maternal hyperhomocysteinemia induces neuroinflammation and neuronal death in the rat offspring cortex. Neurotox Res.

[15] Zhang C, Cai Y, Adachi MT, Oshiro S, Aso T, Kaufman RJ (2001). Homocysteine induces programmed cell death in human vascular endothelial cells through activation of the unfolded protein response. J Biol Chem.

[16] Outinen PA, Sood SK, Pfeifer SI, Pamidi S, Podor TJ, Li J, et al. Homocysteine-induced endoplasmic reticulum stress and growth arrest leads to specific changes in gene expression in human vascular endothelial cells. Blood. 1999;94: 959–967. Available: https://www.ncbi.nlm.nih.gov/pubmed/10419887. 10419887

[17] Althausen S, Paschen W (2000). Homocysteine-induced changes in mRNA levels of genes coding for cytoplasmic- and endoplasmic reticulum-resident stress proteins in neuronal cell cultures. Brain Res Mol Brain Res.

[18] Schröder M, Kaufman RJ (2005). ER stress and the unfolded protein response. Mutat Res.

[19] Walter P, Ron D (2011). The unfolded protein response: from stress pathway to homeostatic regulation. Science.

[20] Urra H, Dufey E, Lisbona F, Rojas-Rivera D, Hetz C (2013). When ER stress reaches a dead end. Biochim Biophys Acta.

[21] Hetz C, Saxena S (2017). ER stress and the unfolded protein response in neurodegeneration. Nat Rev Neurol.

[22] Kaplan P, Tatarkova Z, Sivonova MK, Racay P, Lehotsky J. Homocysteine and Mitochondria in Cardiovascular and Cerebrovascular Systems. Int J Mol Sci. 2020;21. 10.3390/ijms21207698. 10.3390/ijms21207698 PMC7589705 33080955

[23] Folbergrová J, Jesina P, Haugvicová R, Lisý V, Houstek J (2010). Sustained deficiency of mitochondrial complex I activity during long periods of survival after seizures induced in immature rats by homocysteic acid. Neurochem Int.

[24] Fan C-D, Sun J-Y, Fu X-T, Hou Y-J, Li Y, Yang M-F (2017). Astaxanthin attenuates homocysteine-induced cardiotoxicity and by inhibiting mitochondrial dysfunction and oxidative damage. Front Physiol.

[25] Kumar M, Ray RS, Sandhir R (2018). Hydrogen sulfide attenuates homocysteine-induced neurotoxicity by preventing mitochondrial dysfunctions and oxidative damage: In vitro and in vivo studies. Neurochem Int.

[26] Gomez J, Sanchez-Roman I, Gomez A, Sanchez C, Suarez H, Lopez-Torres M (2011). Methionine and homocysteine modulate the rate of ROS generation of isolated mitochondria in vitro. J Bioenerg Biomembr.

[27] Abushik PA, Niittykoski M, Giniatullina R, Shakirzyanova A, Bart G, Fayuk D (2014). The role of NMDA and mGluR5 receptors in calcium mobilization and neurotoxicity of homocysteine in trigeminal and cortical neurons and glial cells. J Neurochem.

[28] Lipton SA, Kim WK, Choi YB, Kumar S, D’Emilia DM, Rayudu PV (1997). Neurotoxicity associated with dual actions of homocysteine at the N-methyl-D-aspartate receptor. Proc Natl Acad Sci U S A.

[29] Zieminska E, Matyja E, Kozlowska H, Stafiej A, Lazarewicz JW (2006). Excitotoxic neuronal injury in acute homocysteine neurotoxicity: role of calcium and mitochondrial alterations. Neurochem Int.

[30] Ganapathy PS, White RE, Ha Y, Bozard BR, McNeil PL, Caldwell RW (2011). The role of N-methyl-D-aspartate receptor activation in homocysteine-induced death of retinal ganglion cells. Invest Ophthalmol Vis Sci.

[31] Yuan J, Lipinski M, Degterev A. Diversity in the Mechanisms of Neuronal Cell Death. Neuron. 2003:401–413. 10.1016/s0896-6273(03)00601-9. 10.1016/s0896-6273(03)00601-9 14556717

[32] Saleem S (2021). Apoptosis, autophagy, necrosis and their multi galore crosstalk in neurodegeneration. Neuroscience.

[33] Levine B, Kroemer G (2008). Autophagy in the pathogenesis of disease. Cell.

[34] Mizushima N, Komatsu M (2011). Autophagy: renovation of cells and tissues. Cell.

[35] Ogata M, Hino S-I, Saito A, Morikawa K, Kondo S, Kanemoto S (2006). Autophagy is activated for cell survival after endoplasmic reticulum stress. Mol Cell Biol.

[36] Tooze SA, Schiavo G (2008). Liaisons dangereuses: autophagy, neuronal survival and neurodegeneration. Curr Opin Neurobiol.

[37] Djavaheri-Mergny M, Maiuri MC, Kroemer G (2010). Cross talk between apoptosis and autophagy by caspase-mediated cleavage of Beclin 1. Oncogene.

[38] Saha A, Saleem S, Paidi RK, Biswas SC (2021). BH3-only proteins Puma and Beclin1 regulate autophagic death in neurons in response to Amyloid-β. Cell Death Discov.

[39] Tripathi M, Zhang CW, Singh BK, Sinha RA, Moe KT, DeSilva DA (2016). Hyperhomocysteinemia causes ER stress and impaired autophagy that is reversed by Vitamin B supplementation. Cell Death Dis.

[40] Khayati K, Antikainen H, Bonder EM, Weber GF, Kruger WD, Jakubowski H (2017). The amino acid metabolite homocysteine activates mTORC1 to inhibit autophagy and form abnormal proteins in human neurons and mice. FASEB J.

[41] Zhao Y, Huang G, Chen S, Gou Y, Dong Z, Zhang X. Homocysteine Aggravates Cortical Neural Cell Injury through Neuronal Autophagy Overactivation following Rat Cerebral Ischemia-Reperfusion. Int J Mol Sci. 2016;17. 10.3390/ijms17081196. 10.3390/ijms17081196 PMC5000594 27455253

[42] Zhang J-W, Yan R, Tang Y-S, Guo Y-Z, Chang Y, Jing L (2017). Hyperhomocysteinemia-induced autophagy and apoptosis with downregulation of hairy enhancer of split 1/5 in cortical neurons in mice. Int J Immunopathol Pharmacol.

[43] Senft D, Ronai ZA (2015). UPR, autophagy, and mitochondria crosstalk underlies the ER stress response. Trends Biochem Sci.

[44] Kaech S, Banker G (2006). Culturing hippocampal neurons. Nat Protoc.

[45] Sato T, Takahoko M, Okamoto H (2006). HuC:Kaede, a useful tool to label neural morphologies in networks in vivo. Genesis.

[46] Rai A, Chatterjee B, Bhowmick S, Sagar S, Roy SS (2020). Beclin 1 controls pigmentation by changing the nuclear localization of melanogenic factor MITF. Biochem Biophys Res Commun.

[47] Roy SS, Madesh M, Davies E, Antonsson B, Danial N, Hajnóczky G (2009). Bad targets the permeability transition pore independent of Bax or Bak to switch between Ca2+-dependent cell survival and death. Mol Cell.

[48] Love MI, Huber W, Anders S (2014). Moderated estimation of fold change and dispersion for RNA-seq data with DESeq2. Genome Biol.

[49] Outinen PA, Sood SK, Pfeifer SI, Pamidi S, Podor TJ, Li J (1999). Homocysteine-induced endoplasmic reticulum stress and growth arrest leads to specific changes in gene expression in human vascular endothelial cells. Blood.

[50] Moore P, El-sherbeny A, Roon P, Schoenlein PV, Ganapathy V, Smith SB (2001). Apoptotic cell death in the mouse retinal ganglion cell layer is induced in vivo by the excitatory amino acid homocysteine. Exp Eye Res.

[51] Austin RC, Lentz SR, Werstuck GH (2004). Role of hyperhomocysteinemia in endothelial dysfunction and atherothrombotic disease. Cell Death Differ.

[52] McCully KS. Chemical Pathology of Homocysteine VI. Aging, Cellular Senescence, and Mitochondrial Dysfunction. Ann Clin Lab Sci. 2018;48: 677–687. Available: https://www.ncbi.nlm.nih.gov/pubmed/30373877. 30373877

[53] Green DR, Kroemer G (2004). The pathophysiology of mitochondrial cell death. Science.

[54] Yu X, Lv J, Zhu Y, Duan L, Ma L (2013). Homocysteine inhibits hepatocyte proliferation via endoplasmic reticulum stress. PLoS One.

[55] Martínez-Pizarro A, Desviat LR, Ugarte M, Pérez B, Richard E (2016). Endoplasmic reticulum stress and autophagy in homocystinuria patients with remethylation defects. PLoS One.

[56] Ozcan U, Yilmaz E, Ozcan L, Furuhashi M, Vaillancourt E, Smith RO (2006). Chemical chaperones reduce ER stress and restore glucose homeostasis in a mouse model of type 2 diabetes. Science.

[57] Cao SS, Kaufman RJ (2014). Endoplasmic reticulum stress and oxidative stress in cell fate decision and human disease. Antioxid Redox Signal.

[58] Bauer TM, Murphy E (2020). Role of mitochondrial calcium and the permeability transition pore in regulating cell death. Circ Res.

[59] Yorimitsu T, Nair U, Yang Z, Klionsky DJ (2006). Endoplasmic reticulum stress triggers autophagy. J Biol Chem.

[60] Hara T, Nakamura K, Matsui M, Yamamoto A, Nakahara Y, Suzuki-Migishima R (2006). Suppression of basal autophagy in neural cells causes neurodegenerative disease in mice. Nature.

[61] Yang Y-P, Ren Y-G, Cai B-Q, Huang D-D (2022). Homocysteine suppresses autophagy through AMPK-mTOR-TFEB signaling in human THP-1 macrophages. J Cardiovasc Pharmacol.

[62] Mizushima N, Yoshimori T, Levine B (2010). Methods in mammalian autophagy research. Cell.

[63] Yang L, Li P, Fu S, Calay ES, Hotamisligil GS (2010). Defective hepatic autophagy in obesity promotes ER stress and causes insulin resistance. Cell Metab.

[64] Antonucci L, Fagman JB, Kim JY, Todoric J, Gukovsky I, Mackey M (2015). Basal autophagy maintains pancreatic acinar cell homeostasis and protein synthesis and prevents ER stress. Proc Natl Acad Sci U S A.

[65] Gukovsky I, Gukovskaya AS. Impaired autophagy triggers chronic pancreatitis: lessons from pancreas-specific atg5 knockout mice. Gastroenterology. 2015:501–505. 10.1053/j.gastro.2015.01.012. 10.1053/j.gastro.2015.01.012 PMC4443443 25613315

[66] Kruger WD (2017). Cystathionine β-synthase deficiency: of mice and men. Mol Genet Metab.

[67] Zatsepina O, Karpov D, Chuvakova L, Rezvykh A, Funikov S, Sorokina S (2020). Genome-wide transcriptional effects of deletions of sulphur metabolism genes in Drosophila melanogaster. Redox Biol.

[68] Postlethwait JH, Yan YL, Gates MA, Horne S, Amores A, Brownlie A (1998). Vertebrate genome evolution and the zebrafish gene map. Nat Genet.

[69] Al-Sadeq DW, Thanassoulas A, Islam Z, Kolatkar P, Al-Dewik N, Safieh-Garabedian B (2022). Pyridoxine non-responsive p.R336C mutation alters the molecular properties of cystathionine beta-synthase leading to severe homocystinuria phenotype. Biochim Biophys Acta Gen Subj.

[70] Kelly PJ, Furie KL, Kistler JP, Barron M, Picard EH, Mandell R (2003). Stroke in young patients with hyperhomocysteinemia due to cystathionine beta-synthase deficiency. Neurology.

[71] Smith AD, Refsum H (2016). Homocysteine, B Vitamins, and Cognitive Impairment. Annu Rev Nutr.

[72] Zhang T, Huang D, Hou J, Li J, Zhang Y, Tian M (2020). High-concentration homocysteine inhibits mitochondrial respiration function and production of reactive oxygen species in neuron cells. J Stroke Cerebrovasc Dis.

[73] Lam M, Marsters SA, Ashkenazi A, Walter P. Misfolded proteins bind and activate death receptor 5 to trigger apoptosis during unresolved endoplasmic reticulum stress. Elife. 2020;9. 10.7554/eLife.52291. 10.7554/eLife.52291 PMC7041945 31904339

[74] Pohl C, Dikic I (2019). Cellular quality control by the ubiquitin-proteasome system and autophagy. Science.

[75] Maday S (2016). Mechanisms of neuronal homeostasis: autophagy in the axon. Brain Res.

[76] Nixon RA, Yang D-S. Autophagy and neuronal cell death in neurological disorders. Cold Spring Harb Perspect Biol. 2012;4. 10.1101/cshperspect.a008839. 10.1101/cshperspect.a008839 PMC3475163 22983160

[77] Wang M, Liang X, Cheng M, Yang L, Liu H, Wang X (2019). Homocysteine enhances neural stem cell autophagy in in vivo and in vitro model of ischemic stroke. Cell Death Dis.

[78] Koike M, Shibata M, Tadakoshi M, Gotoh K, Komatsu M, Waguri S (2008). Inhibition of autophagy prevents hippocampal pyramidal neuron death after hypoxic-ischemic injury. Am J Pathol.

[79] Watanabe M, Osada J, Aratani Y, Kluckman K, Reddick R, Malinow MR (1995). Mice deficient in cystathionine beta-synthase: animal models for mild and severe homocyst(e)inemia. Proc Natl Acad Sci U S A.

[80] Maclean KN, Sikora J, Kožich V, Jiang H, Greiner LS, Kraus E (2010). Cystathionine beta-synthase null homocystinuric mice fail to exhibit altered hemostasis or lowering of plasma homocysteine in response to betaine treatment. Mol Genet Metab.

[81] Ars CL, Nijs IM, Marroun HE, Muetzel R, Schmidt M, Steenweg-de Graaff J (2019). Prenatal folate, homocysteine and vitamin B levels and child brain volumes, cognitive development and psychological functioning: the Generation R Study. Br J Nutr.

[82] Shcherbitskaia AD, Vasilev DS, Milyutina YP, Tumanova NL, Mikhel AV, Zalozniaia IV, et al. Prenatal Hyperhomocysteinemia Induces Glial Activation and Alters Neuroinflammatory Marker Expression in Infant Rat Hippocampus. Cells. 2021;10. 10.3390/cells10061536. 10.3390/cells10061536 PMC8234222 34207057

[83] Heo Y-A (2022). Sodium Phenylbutyrate and Ursodoxicoltaurine: First Approval. CNS Drugs.

[84] Velazquez R, Ferreira E, Winslow W, Dave N, Piras IS, Naymik M (2020). Maternal choline supplementation ameliorates Alzheimer’s disease pathology by reducing brain homocysteine levels across multiple generations. Mol Psychiatry.

[85] Kumar M, Mahajan A, Sapehia D, Kaur J, Sandhir R (2019). Effects of altered maternal folate and vitamin B on neurobehavioral outcomes in F1 male mice. Brain Res Bull.

