# Supplementary material for: Defective quality control autophagy in Hyperhomocysteinemia promotes ER stress and consequent neuronal apoptosis through proteotoxicity
Source: Cell Commun Signal. 2023 Sep 25;21:258. doi: 10.1186/s12964-023-01288-w (PMC10518934; doi:10.1186/s12964-023-01288-w)
Supplement: Supplementary file 2 — Additional file 1. [file 12964_2023_1288_MOESM1_ESM.pdf]

Figure S1

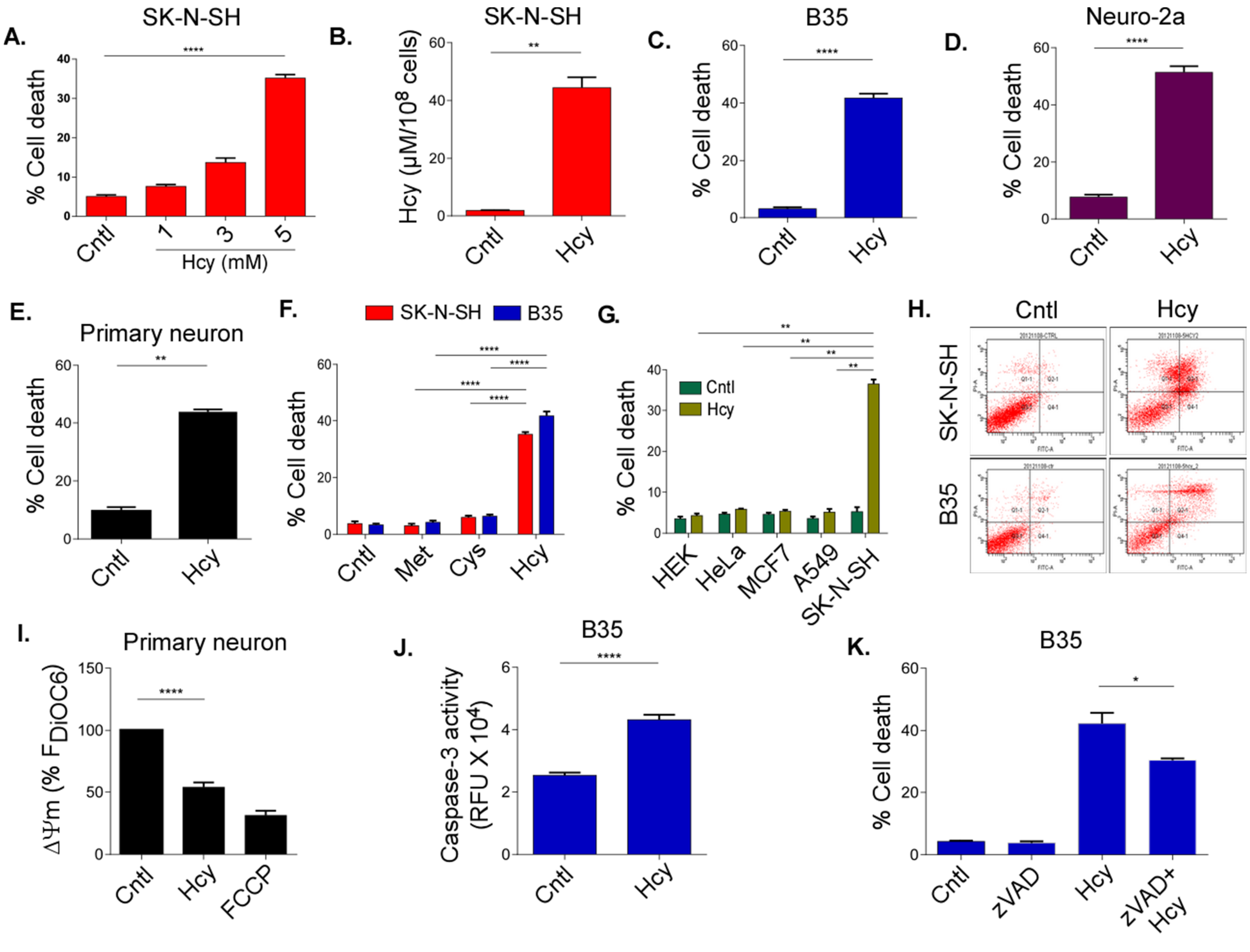
A.
SK-N-SH
% Cell death
Cntl
1
3
5
Hcy (mM)
B.
SK-N-SH
Hcy (μM/10^8 cells)
Cntl
Hcy
C.
B35
% Cell death
Cntl
Hcy
D.
Neuro-2a
% Cell death
Cntl
Hcy
E.
Primary neuron
% Cell death
Cntl
Hcy
F.
SK-N-SH
B35
% Cell death
Cntl
Met
Cys
Hcy
G.
Cntl
Hcy
% Cell death
HEK
HeLa
MCF7
A549
SK-N-SH
H.
Cntl
Hcy
SK-N-SH
B35
I.
Primary neuron
ΔΨm (% F DiOC6)
Cntl
Hcy
FCCP
J.
B35
Caspase-3 activity (RFU X 10^4)
Cntl
Hcy
K.
B35
% Cell death
Cntl
zVAD
Hcy
zVAD+ Hcy

Figure S1. (**A**) Bar graph represents percentage of cell death by increasing concentration of Hcy treatment in SK-N-SH cells. (**B**) Bar diagram of intracellular Hcy concentration in SK-N-SH cells (normalized to number of cells) post 5 mM Hcy treatment for 24 h as determined by HPLC. (**C**), (**D**) and (**E**) Represents percentage of cell death in Hcy treated B35 (rat neuroblastoma), Neuro-2a (mouse neuroblastoma) and rat primary cortical neurons respectively. (**F**) Bar graph represents percentage of cell death upon 5 mM Methionine and Cysteine treatment (24 h) compared to Hcy in B35 and SK-N-SH cells. (**G**) Bars showing percent cell death upon Hcy treatment in HEK (kidney origin), HELA (cervix origin), MCF7 (breast origin) and A549 (lung origin) compared to SK-N-SH cells. (**H**) Representative flow cytometric plots of Annexin V-FITC/PI staining for detection of apoptotic cells. (**I**) Bars showing loss of mitochondrial membrane potential ($\Delta\Psi m$) as measured by potentiometric dye DiOC6 in Hcy treated primary neurons. FCCP was used as a positive control. (**J**) Bars represent excess caspase-3 enzyme activity (measured by AcDEVD substrate) in Hcy treated B35 cells. (**K**) Bar diagram represents rescue of Hcy-induced toxicity by a pan-caspase inhibitor (zVAD-FMK) in B35 cells. Concentration of Hcy was 5mM for cell lines and 0.75mM for primary neurons. Data are shown as Mean±SEM with $n \geq 3$. *$P < 0.05$. **$P < 0.01$. ****$P < 0.0001$.

Figure S2

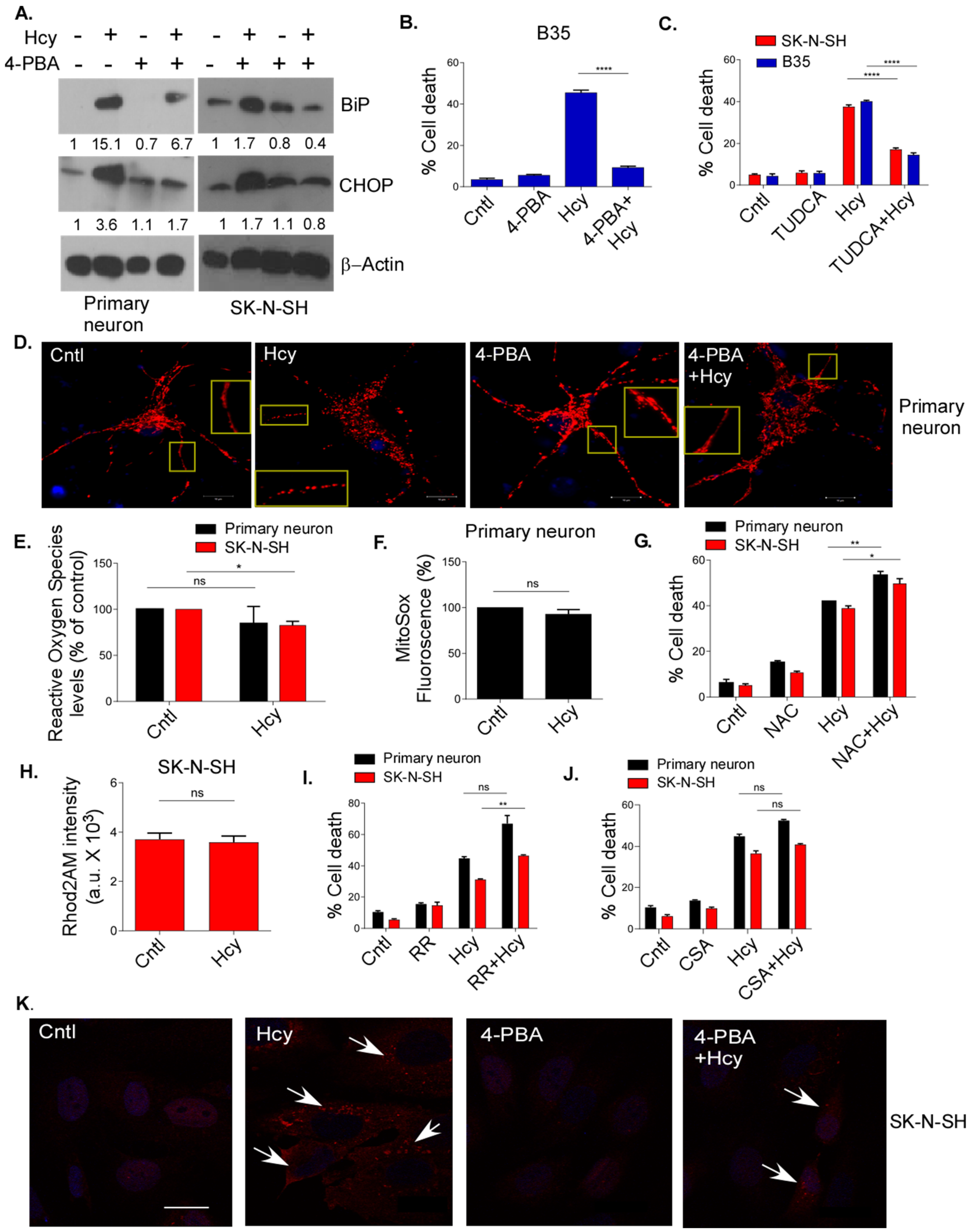
A.
Hcy - + - + - + - +
4-PBA - - + + - + + +
BiP
1 15.1 0.7 6.7 1 1.7 0.8 0.4
CHOP
1 3.6 1.1 1.7 1 1.7 1.1 0.8
β–Actin
Primary neuron
SK-N-SH
B.
B35
% Cell death
Cntl
4-PBA
Hcy
4-PBA+ Hcy
****
C.
SK-N-SH
B35
% Cell death
Cntl
TUDCA
Hcy
TUDCA+Hcy
D.
Cntl
Hcy
4-PBA
4-PBA +Hcy
Primary neuron
E.
Primary neuron
SK-N-SH
Reactive Oxygen Species levels (% of control)
ns
Cntl
Hcy
F.
Primary neuron
MitoSox Fluoroscence (%)
ns
Cntl
Hcy
G.
Primary neuron
SK-N-SH
% Cell death
Cntl
NAC
Hcy
NAC+Hcy
H.
SK-N-SH
Rhod2AM intensity (a.u. X 10³)
ns
Cntl
Hcy
I.
Primary neuron
SK-N-SH
% Cell death
ns
Cntl
RR
Hcy
RR+Hcy
J.
Primary neuron
SK-N-SH
% Cell death
ns
Cntl
CSA
Hcy
CSA+Hcy
K.
Cntl
Hcy
4-PBA
4-PBA +Hcy
SK-N-SH

Figure S2. (**A**) Immunoblots showing protective effect of 4-PBA on ER stress markers in Hcy treated primary neurons (left) and SK-N-SH cells (right). β-actin was used as a loading control. (**B**) Bars represent the protective effect of 4-PBA on Hcy induced cell death in B35 cells. (**C**) Bar diagram showing protective effect of ER stress blocker TUDCA on Hcy induced cell death in B35 and SK-N-SH cells. (**D**) Representative confocal images showing protection of mitochondrial fragmentation by 4-PBA in Hcy treated primary neurons. Scale bar 10µm. Bars showing no significant increase in total reactive oxygen species level as measured by DCFDA (**E**) and mitochondrial reactive oxygen species level as measured by MitoSox (**F**) in Hcy-treated neuronal cells. (**G**) Bars showing no protective effect of the antioxidant N-acetyl cysteine (NAC) on Hcy-induced cell death in primary neurons and SK-N-SH cells. (**H**) Mitochondrial calcium level measurement in Hcy-treated SK-N-SH cells using RHOD-2AM probe showing no significant increase in $[Ca^{2+}]_m$. Bar diagrams showing no protection by mitochondrial uniporter blocker Ruthenium Red (RR) (**I**) or PTP blocker Cyclosporine A (CSA) (**J**) on Hcy induced cell death in primary neurons and SK-N-SH cells. (**K**) Immunocytochemistry analysis showing protective effect of 4-PBA on polyubiquitinated protein aggregates induced by Hcy in SK-N-SH cells. White arrows showing aggregated protein puncta. Confocal images with a scale bar of 20µm. Concentration of Hcy was 5mM for cell lines and 0.75mM for primary neurons. Data are shown as Mean±SEM with n ≥ 3. *P < 0.05. **P < 0.01. ****P < 0.0001. NS – non significant.

Figure S3

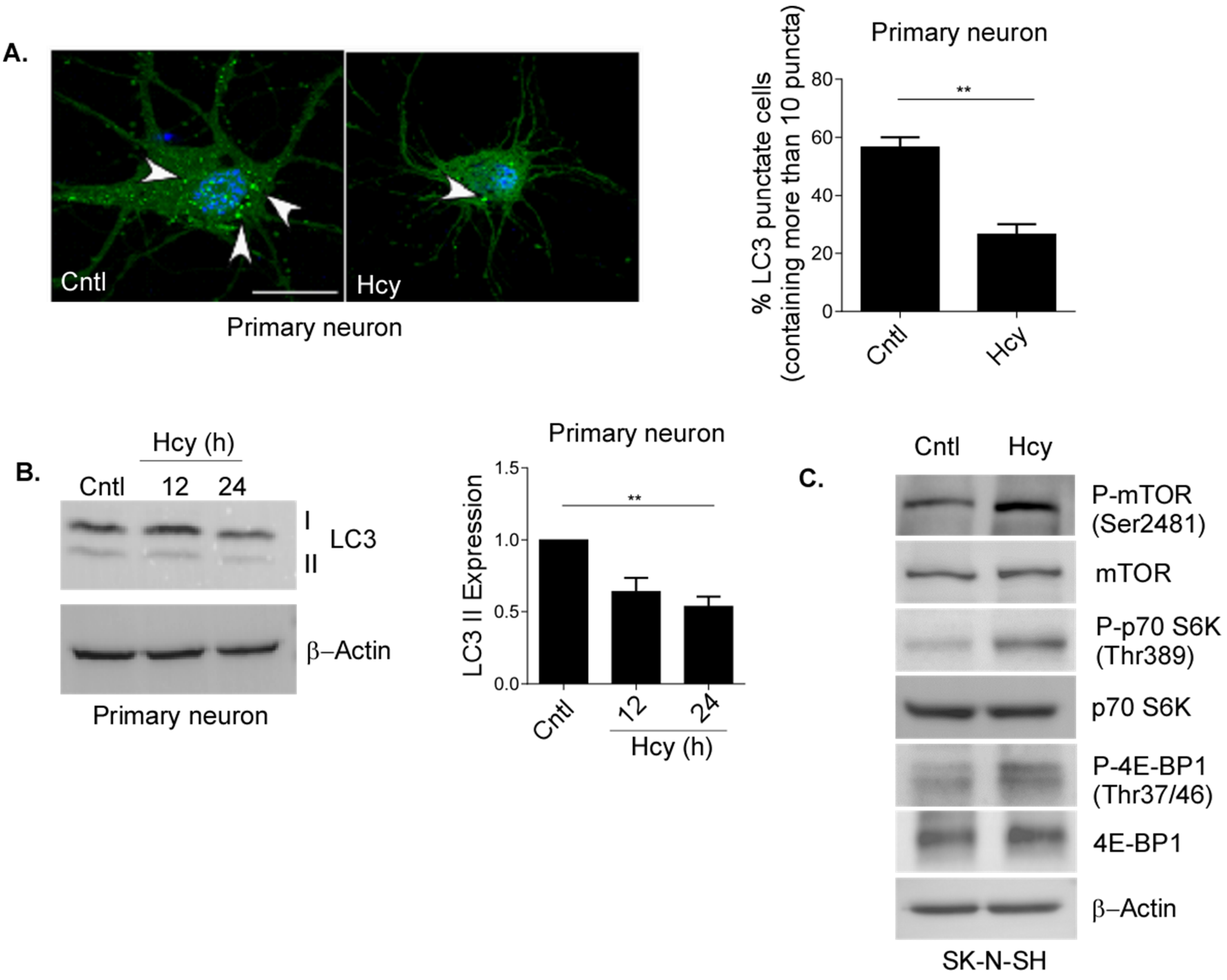
A.
Cntl
Hcy
Primary neuron
Primary neuron
% LC3 punctate cells (containing more than 10 puncta)
80
60
40
20
0
**
Cntl
Hcy
B.
Hcy (h)
Cntl
12
24
I
II
LC3
β–Actin
Primary neuron
Primary neuron
LC3 II Expression
1.5
1.0
0.5
0.0
**
Cntl
12
24
Hcy (h)
C.
Cntl
Hcy
P-mTOR (Ser2481)
mTOR
P-p70 S6K (Thr389)
p70 S6K
P-4E-BP1 (Thr37/46)
4E-BP1
β–Actin
SK-N-SH

Figure S3. (**A**) Left panel: representative confocal images showing LC3-GFP puncta in primary neurons transiently transfected with LC3-GFP construct. Arrow heads represent LC3 GFP puncta. Scale bar 20µm. Right panel: bar graphs showing percent quantitation of LC3 GFP puncta containing cells (≥ 10 puncta per cell). (**B**) Immunoblot (left panel) showing a time dependent decrease in LC3-II form (marker of autophagy) and the bars (right panel) showing the densitometric quantitation of lipidated LC3 II level in Hcy-treated neurons. β-actin was used as a loading control in immunoblot. (**C**) Immunoblot analysis of mTOR signaling proteins (mTOR, p70 S6K, and 4E-BP1) showing activation of mTOR pathway and its downstream targets as the phosphorylation increased in these proteins upon Hcy treatment in SK-N-SH cells. β-actin was used as a loading control. Concentration of Hcy was 5mM for SK-N-SH cells and 0.75mM for primary neurons. Data are shown as Mean±SEM with $n \geq 3$. $^{**}P < 0.01$.

Figure S4

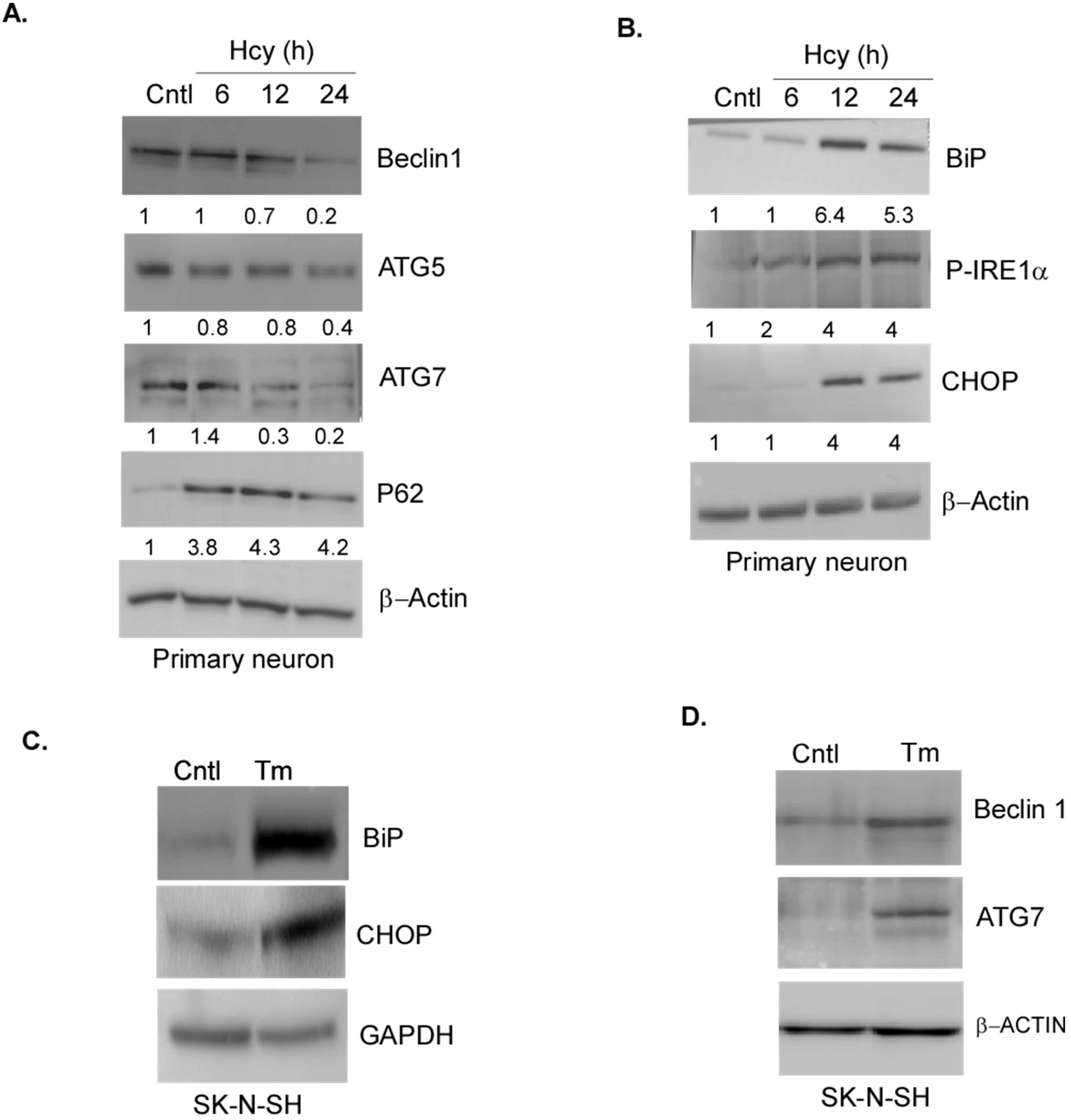
A.
Hcy (h)
Cntl 6 12 24
Beclin1
1 1 0.7 0.2
ATG5
1 0.8 0.8 0.4
ATG7
1 1.4 0.3 0.2
P62
1 3.8 4.3 4.2
β–Actin
Primary neuron
B.
Hcy (h)
Cntl 6 12 24
BiP
1 1 6.4 5.3
P-IRE1α
1 2 4 4
CHOP
1 1 4 4
β–Actin
Primary neuron
C.
Cntl Tm
BiP
CHOP
GAPDH
SK-N-SH
D.
Cntl Tm
Beclin 1
ATG7
β–ACTIN
SK-N-SH

Figure S4. Immunoblots of same cell lysates showing a time dependent suppression of autophagy (**A**) and an activation of ER stress (**B**) by Hcy in SK-N-SH cells. β-actin was used as a loading control. Immunoblots of same cell lysates showing the activation of both ER stress markers (**C**) and autophagic proteins (**D**) by a known ER stress generator Tunicamycin (Tm) in SK-N-SH cells. β-actin and GAPDH were used as loading controls respectively. Concentration of Hcy was 5mM for SK-N-SH cells and 0.75mM for primary neurons. Data are shown as Mean±SEM with $n \geq 3$.

Figure S5

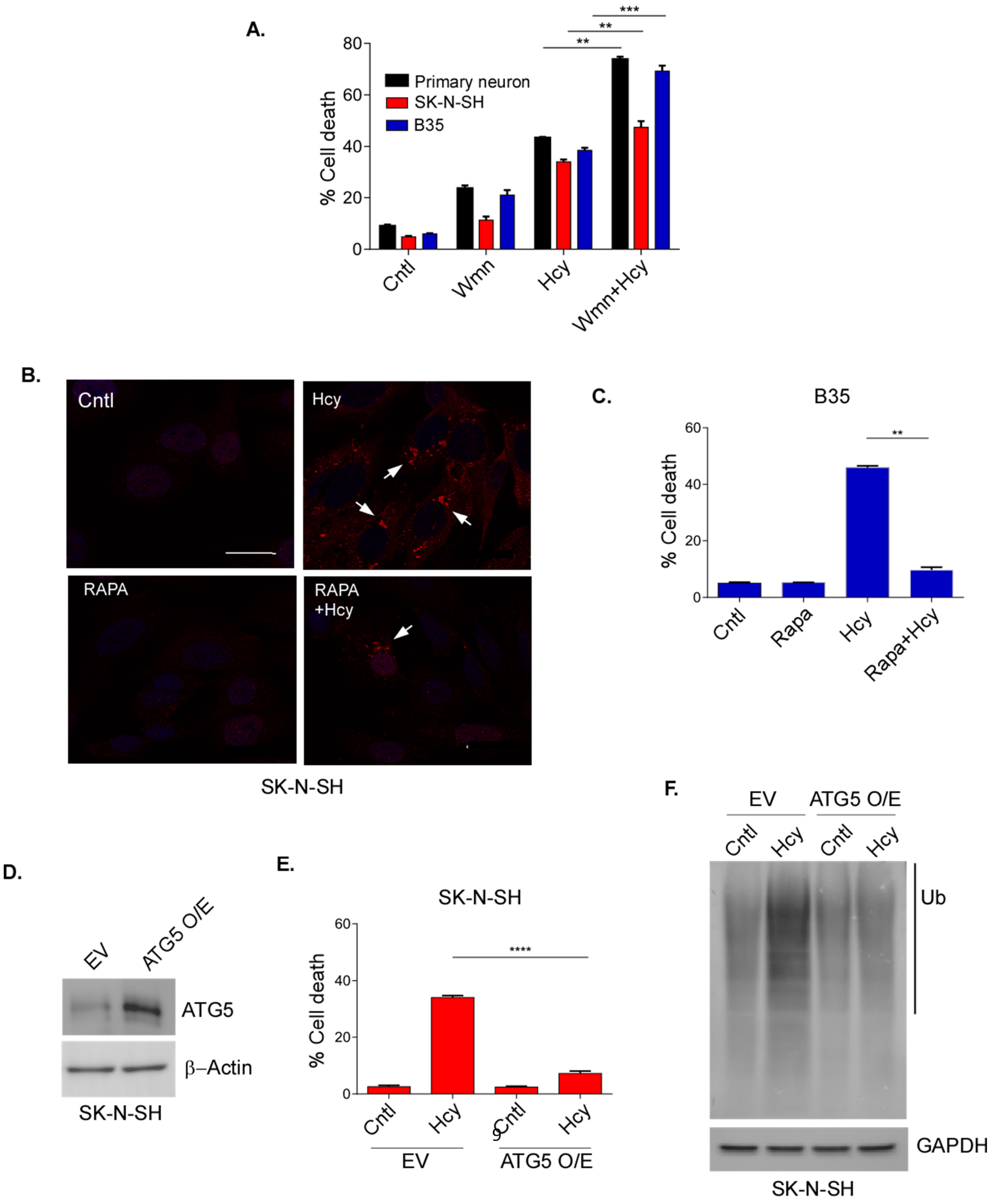
A.
***
**
**
80
60
40
20
0
% Cell death
Primary neuron
SK-N-SH
B35
Cntl
Wmn
Hcy
Wmn+Hcy
B.
Cntl
Hcy
RAPA
RAPA +Hcy
SK-N-SH
C.
B35
60
40
20
0
% Cell death
**
Cntl
Rapa
Hcy
Rapa+Hcy
D.
EV
ATG5 O/E
ATG5
β–Actin
SK-N-SH
E.
SK-N-SH
60
40
20
0
% Cell death
****
Cntl
Hcy
Cntl
Hcy
EV
ATG5 O/E
F.
EV
ATG5 O/E
Cntl
Hcy
Cntl
Hcy
Ub
GAPDH
SK-N-SH

Figure S5. (**A**) Bar diagram showing increase in Hcy-induced cell death in presence of an early autophagic blocker Wortmannin (Wmn) in neuronal cells. (**B**) Immunocytochemical images showing the lowering of polyubiquitinated protein puncta by RAPA in Hcy-treated SK-N-SH cells. White arrows indicating aggregated protein puncta. Confocal images with a scale bar of 20µm. C) Bars represent the protective effect of RAPA on Hcy induced cell death in B35 cells. (**D**) Immunoblot confirming higher expression of ATG5 protein in SK-N-SH cells transiently overexpressing ATG5. GAPDH was used as a loading control. ATG5 overexpression (constitutively activated autophagy) protects against cell death (**E**) as well as lowers the level of polyubiquitinated proteins (**F**) compared to the vector control SK-N-SH cells upon Hcy treatment. GAPDH was used as a loading control for the immunoblot. Concentration of Hcy was 5mM for cell lines and 0.75mM for primary neurons. Data are shown as Mean±SEM with $n \geq 3$. $^{**}P < 0.01$. $^{***}P < 0.001$.$^{****}P < 0.0001$

Figure S6

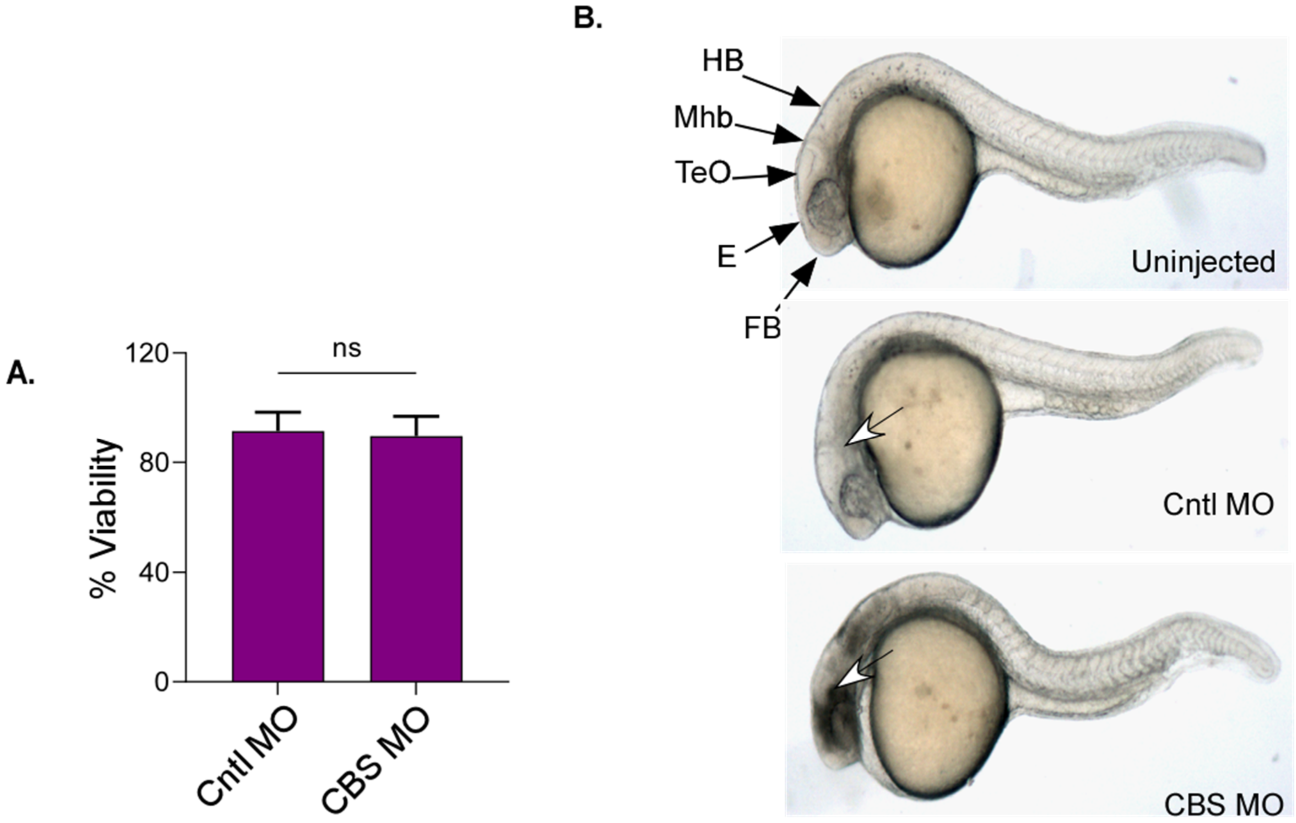

HB — Hindbrain
Mhb — Midbrain hindbrain boundry
TeO — Optic tectum
E — Eye
FB — Forebrain

Figure S6. (**A**) Bar diagram showing percentage of viability in control and CBS morpholino injected embryos at 24 hpf. confirming no significant toxicity of CBS downregulation. (**B**) Representative images showing the effect of CBS knockdown in the brain region of CBS morphants compared to control and uninjected embryos. White arrows indicate damaged brain regions in CBS morphants, largely in the region of midbrain hindbrain boundary. (**C**) Bar diagram represents fold change in the mRNA level of Beclin1 in CBS morphants compared to controls. Data are shown as Mean±SEM with $n \geq 3$. NS – non significant.
